# Supplementary material for: Intraspecific and intraindividual trait variability decrease with tree richness in a subtropical tree biodiversity experiment
Source: Nat Commun. 2025 Dec 11;16:11009. doi: 10.1038/s41467-025-67265-8 (PMC12698814; doi:10.1038/s41467-025-67265-8)
Supplement: Supplementary file 1 — Supplementary Information [file 41467_2025_67265_MOESM1_ESM.pdf]

Supplementary Information for

Intraspecific and intraindividual trait variability decrease with tree richness in a subtropical tree diversity experiment

Pablo Castro Sánchez-Bermejo[1, 2, 3*], Carlos Pérez Carmona[4, 5], Meredith Christine Schuman[6, 7], Raquel Benavides[8, 9], Lena Sachsenmaier[2, 10], Shan Li[11], Xiaojuan Liu[11], Sylvia Haider[3]

1. Martin Luther University Halle-Wittenberg, Institute of Biology/Geobotany and Botanical Garden, Halle (Saale), Germany

2. German Centre for Integrative Biodiversity Research (iDiv) Halle-Jena-Leipzig, Leipzig, Germany

3. Leuphana University of Lüneburg, School of Sustainability, Institute of Ecology, Lüneburg, Germany

4. Misión Biológica de Galicia (MBG), Consejo Superior de Investigaciones Científicas (CSIC), Pontevedra, Spain

5. Department of Botany, Institute of Ecology and Earth Sciences, University of Tartu, Tartu, Estonia

6. Department of Geography, Faculty of Science, University of Zurich, Zurich, Switzerland

7. Department of Chemistry, Faculty of Science, University of Zurich, Zurich, Switzerland

8. Department of Natural Systems and Resources, ETSI Montes, Forestal y del Medio Natural, Universidad Politécnica de Madrid, Madrid, Spain

9. Center for the Biodiversity Conservation and Sustainable Development. CBDS-UPM, Madrid, Spain

10. Systematic Botany and Functional Biodiversity, Leipzig University, Leipzig, Germany

11. State Key Laboratory of Vegetation and Environmental Change, Institute of Botany, Chinese Academy of Sciences, Beijing, China

*Corresponding author. Email: pablokstrosb@gmail.com

SUPPORTING INFORMATION

Supplementary Fig. 1. Expected patterns along the experimental tree species richness gradient for two alternative hypotheses.

Supplementary Fig. 2. Regression coefficients for the effects of tree species richness on the intraspecific and intraindividual variability on five leaf functional traits and two main axes of leaf trait variability.

Supplementary Fig. 3. Results of a principal component analysis (PCA) of five leaf functional traits belonging to eight different tree species.

Supplementary Fig. 4. Location of eight sampled species in a functional trait space assessed by a principal component analysis (PCA) for five leaf functional traits.

Supplementary Fig. 5. Regression coefficients for the effects of tree species richness on the intraspecific and intraindividual spectral variability on 29 principal components associated with segments of the leaf reflectance spectrum.

Supplementary Fig. 6. Conceptual framework for the segmentation of the leaf reflectance spectrum using the Hierarchical Spectral Clustering with Parallel Analysis (HSC-PA).

Supplementary Fig. 7. Segmentation of leaf reflectance spectrum obtained using Hierarchical Spectral Clustering with Parallel Analysis (HSC-PA).

Supplementary Fig. 8. Conceptual model representing the relationships between variables that could affect intraspecific overlap in leaf functional traits.

Supplementary Fig. 9. Effect of tree species richness on intraspecific overlap.

Supplementary Fig. 10. Results of non-simplified piecewise structural equation models (SEM) studying the mechanisms driving the intraspecific overlap in leaf functional traits.

Supplementary Fig. 11. Conceptual framework for measuring community functional diversity based on individual leaf trait values (following the approach of Carmona et al.[39]).

Supplementary Fig. 12. Conceptual framework for the null model approach based on the randomization of different sources of variation.

Supplementary Fig. 13. Results of linear mixed-effects models to test the joint effect of tree species richness and the type of null models on standardized effect sizes (SES) of two univariate functional indices (functional richness (FRic) and functional divergence (FDiv)) calculated from the two main axes of leaf variation (PC1 and PC2) and for four different sources of trait variation.

Supplementary Fig. 14. Description associations between different regions of the leaf reflectance spectrum and biochemical and structural components of leaves (adapted from Li et al.[64]).

Supplementary Fig. 15. Bar plots for the variance partitioning of leaf variation.

Supplementary Fig. 16. Location and slope of the sampled trees within the experimental site.

Supplementary Fig. 17. Spatial arrangement of sampled trees within each sampled plot.

Supplementary Fig. 18. Differences in spatial distances between trees along the diversity gradient and among plots.

Supplementary Fig. 19. Analytical workflow used to generate a trait matrix from spectral data and two calibration sets.

Supplementary Fig. 20. Leaf reflectance spectra for the eight study species.

Supplementary Fig. 21. Distribution of trait values of the leaf economics spectrum and stomata calibration sets for each species.

Supplementary Fig. 22. Scatter plot of predicted and measured trait values in the test and the train samples.

Supplementary Fig. 23. Evolution of the error during the training of convolutional neural networks for trait prediction.

Supplementary Fig. 24. Bar plot and heatmap of the distribution of missing trait data in the leaf-level dataset.

Supplementary Fig. 25. Excluded values from predicted leaf-level data for seven leaf functional traits.

Supplementary Fig. 26. Coefficient of determination (R^2) calculated under different scenarios of completeness of the training set.

Supplementary Fig. 27. Analytical framework used to assess the metrics of intraindividual variability, intraspecific variability and intraspecific overlap.

Supplementary Fig. 28. Differences in aspect among sampled trees.

Supplementary Fig. 29. Evolution of mean and variance of simulated values of FRic and FDiv from different null models with increasing number of randomizations.

Supplementary Table 1. Summary of a principal component analysis for five leaf functional traits, including loadings, standard deviation, proportion of the variance explained by each component and the adjusted eigenvalue obtained in a Horn's parallel analysis.

Supplementary Table 2. Results for linear mixed-effects models studying the effects of tree species richness on multivariate functional indices used to estimate intraspecific variability, intraindividual variability and intraspecific overlap.

Supplementary Table 3. Species included in the study.

Supplementary Table 4. Results for linear mixed-effects models studying the effects of tree species richness on functional indices used to estimate intraspecific variability, intraindividual variability in 30 principal components associated to segments of the leaf reflectance spectrum.

Supplementary Table 5. Results for linear mixed-effects models studying the effects of tree species richness and type of null model on standardized effect sizes of two functional indices.

Supplementary Table 6. Coefficient of determination (R^2) and root mean squared error (RMSE) for each of the eight species included in the study.

Supplementary Table 7. Layers and hyperparameters used for building a convolutional neural network for every trait, and coefficient of determination (R^2) and root mean squared error (RMSE) for the test and the train samples.

Supplementary Table 8. Distribution of missing trait data in the leaf-level dataset across species and traits.

Supplementary Table 9. Competing models to identify the drivers of intraspecific and intraindividual trait variability.

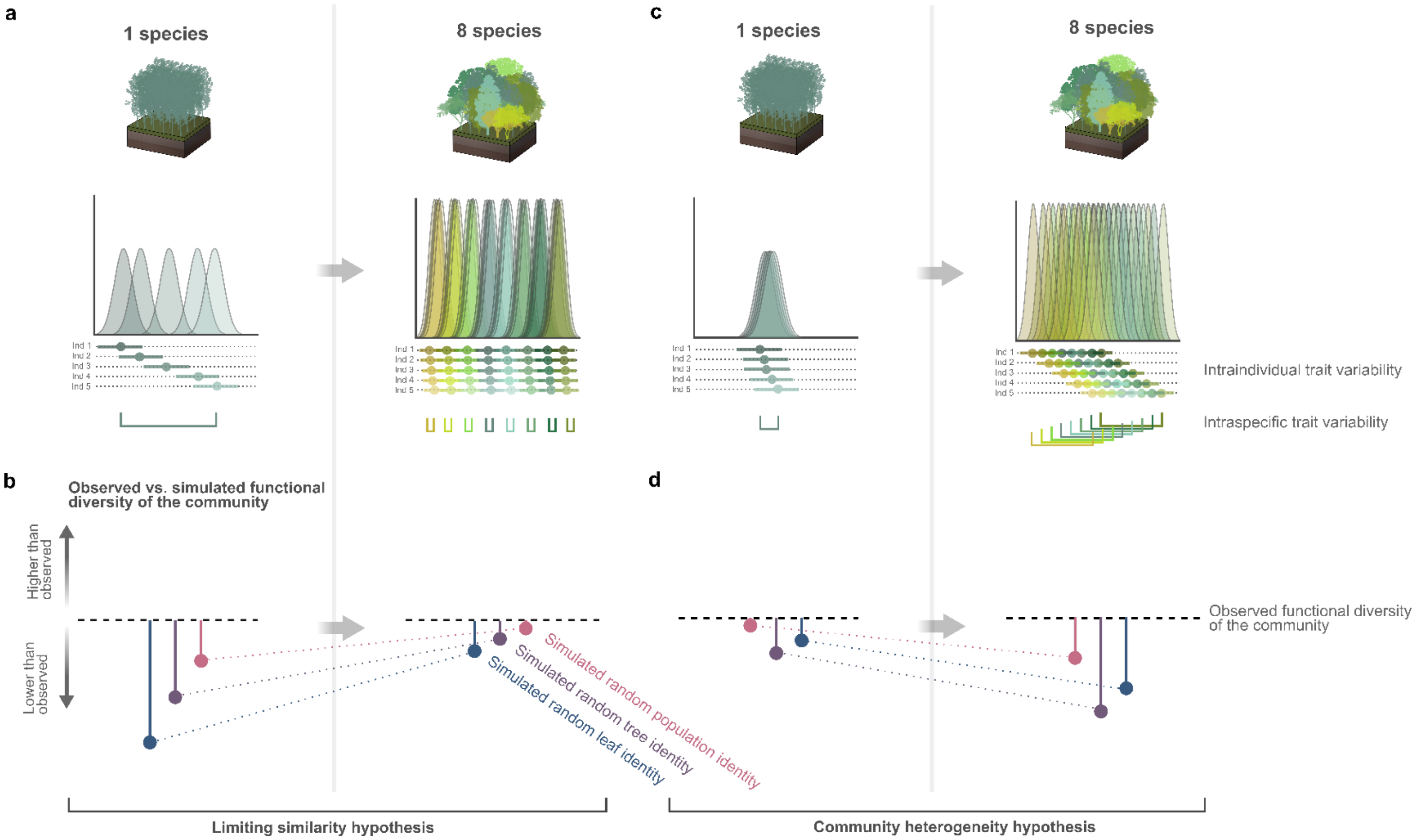

Supplementary Fig. 1. Expected patterns along the experimental tree species richness gradient for two alternative hypotheses. In the case of the limiting similarity hypothesis, along the tree species richness gradient, we expect (**a**) a reduction of intraspecific trait variability (i.e. differences between the mean trait values of individual trees) and of intraindividual trait variability (i.e. differences between the trait values within a tree; represented as error bars around points of individual mean trait values), which would result in increasing intraspecific overlap (i.e. shared trait space between trees belonging to the same population), while (**c**) an increase in intraspecific trait variability (but still a decrease in intraindividual trait variability as supported by previous literature[16, 32]) is expected to occur in the case of the community heterogeneity hypothesis. Curves represent the trait distributions of populations, with inner stacked curves belonging to the trait distribution of tree individuals. The structure of trait variation within species can influence community functional diversity and that is why (**b**) we expect functional diversity in observed communities (represented as a baseline with a grey dashed line) to be higher compared to the functional diversity of virtual assemblages (colored values) for which different sources of trait variation have been randomized in the case of the limiting similarity hypothesis. Specifically, we expect that the functional diversity of observed communities would be more similar to those assessed with models that randomize the identity of the populations (pink points) compared to those assessed with models that randomize the identity of the trees (purple points), and the total pool of leaves within a species (blue points), respectively. In addition, we expect these differences to be higher with low tree species richness due to the importance of intraspecific and intraindividual trait variability in the functional diversity of species-poor communities. In contrast, (**d**) for the community heterogeneity hypothesis we expect an increase in the deviations from the null model with tree species richness, and that the observed functional diversity will be especially dissimilar to those assessed with models that randomize the identity of the trees (purple points).

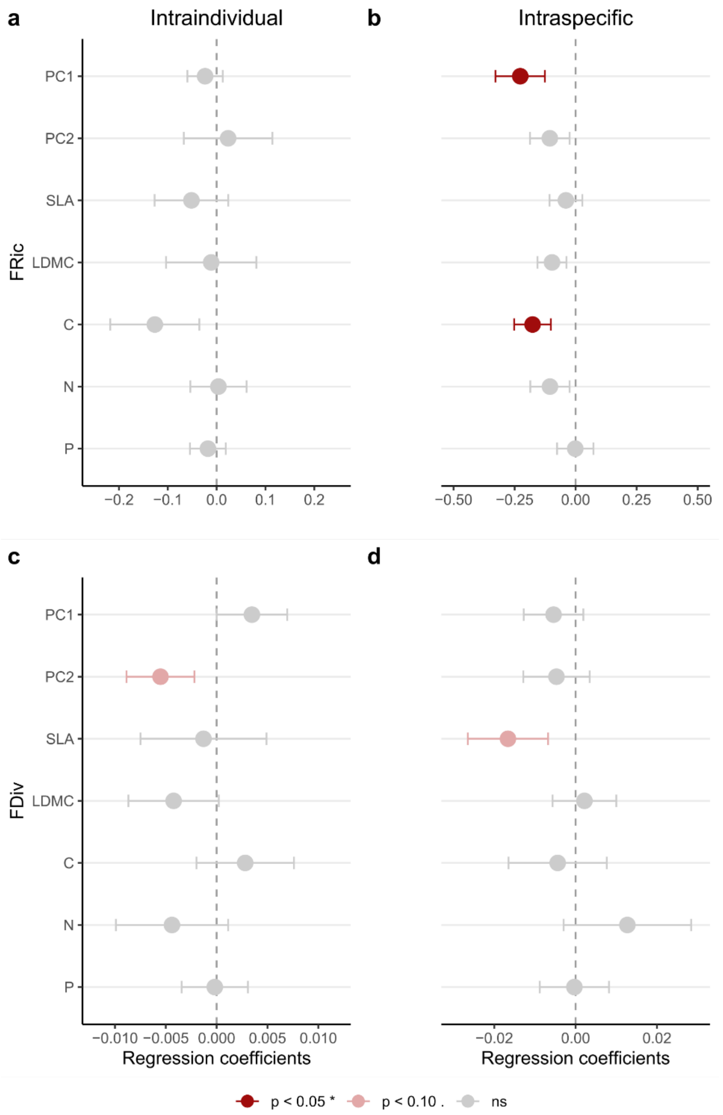

Supplementary Fig. 2. Regression coefficients for the effects of tree species richness on the intraspecific and intraindividual variability on five leaf functional traits and two main axes of leaf trait variability. Regression coefficients for the effects of tree species richness on the intraspecific (N = 63) and intraindividual (N = 63) variability on five leaf functional traits and two main axes of leaf trait variability. The effects of tree species richness on intraspecific and intraindividual variability were studied for five functional traits (specific leaf area (SLA), leaf dry matter content (LDMC), leaf carbon content (C), leaf nitrogen content (N) and leaf phosphorus content (P) and for two main axes of leaf trait variability (PC1 and PC2). Significant negative effects of tree species richness were found in the case intraspecific FRic for PC1 (χ^2(df = 1) = 4.85, P = 0.03, standard estimate (β) = -0.28) and C (χ^2(df = 1) = 5.21, P = 0.02, β = -0.32), while there was a marginally significant effect on the intraspecific FDiv for SLA (χ^2(df = 1) = 3.70, P = 0.05, β = -0.30). In the case of intraindividual trait variability, only the FDiv for PC2 showed a marginally significant decrease with tree species richness (χ^2(df = 1) = 2.74, P = 0.09, β = -0.09). Colors represent the significance as determined by a likelihood ratio test against a model with no tree species richness effect. (red $p < 0.05$, pink < 0.01, grey $p > 0.05$). Error bars correspond to the standard error.

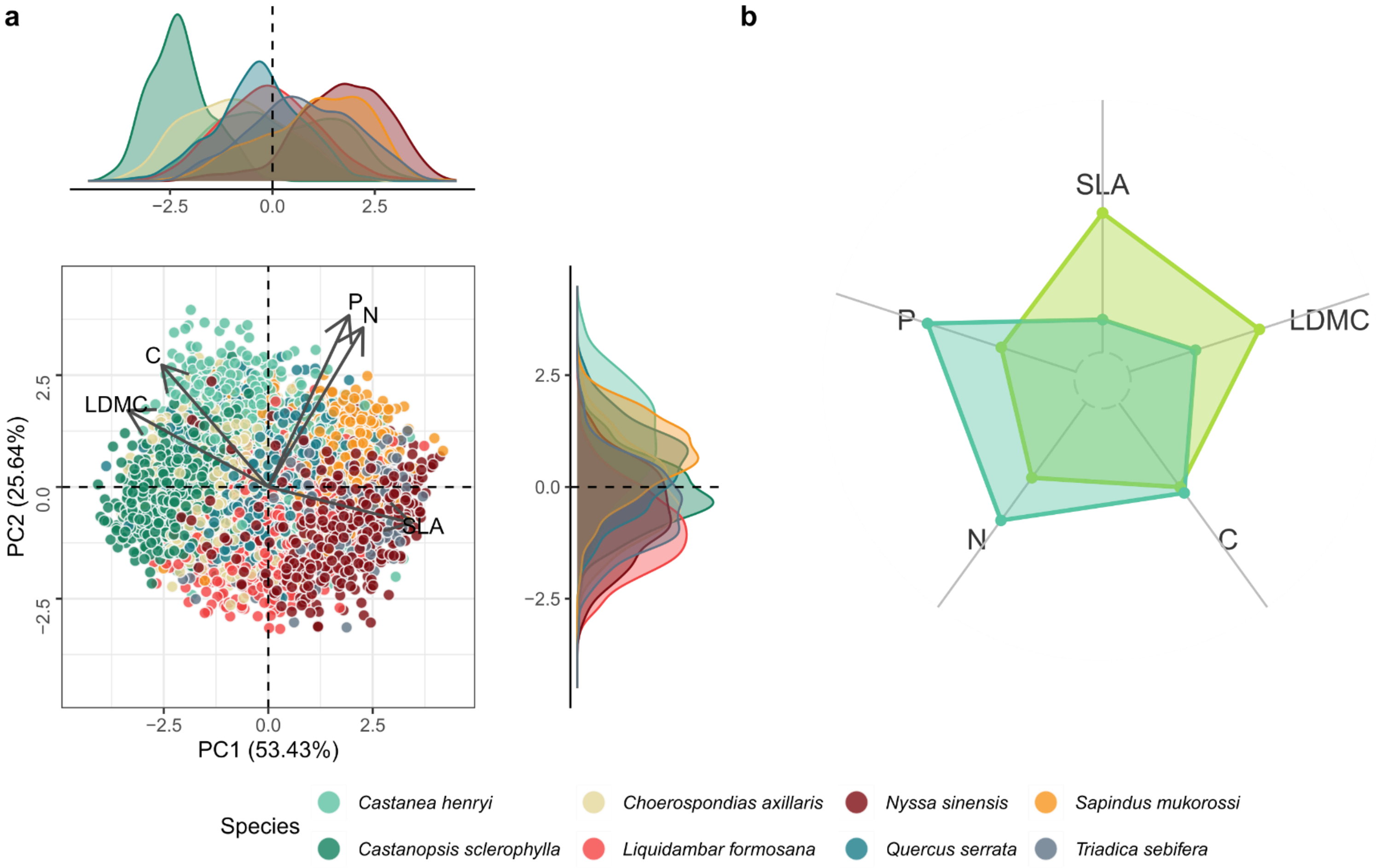

Supplementary Fig. 3. Results of a principal component analysis (PCA) of five leaf functional traits belonging to eight different tree species. (**a**) Main axes of a principal component analysis, including the location for every leaf and arrows representing the eigenvalues of every trait in the PCA axes, and (**b**) radar plot representing the eigenvalues of the traits in the two main axes. The first axis represents the variation in growth strategy, with lower values associated with a conservative strategy while higher values correspond to an acquisitive strategy. The second axis mainly associates with leaf P and N.

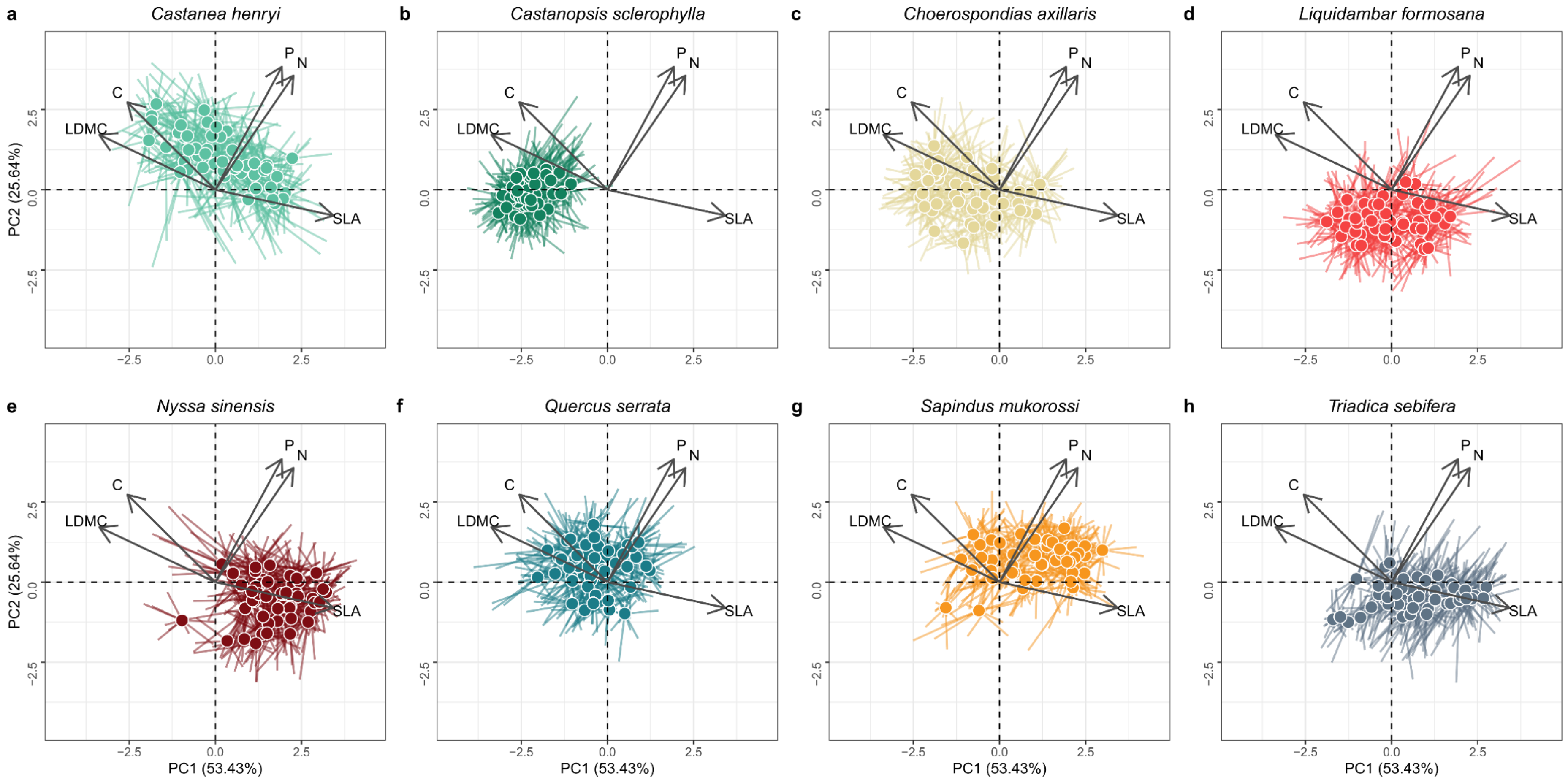

Supplementary Fig. 4. Location of eight sampled species in a functional trait space assessed by a principal component analysis (PCA) for five leaf functional traits. Each panel corresponds to a different species, dots represent the mean trait values of every tree (centroid) and lines connect the centroid to the individual values of the leaves belonging to the tree. The figure suggests that there are differences between species in terms of their leaf strategy (e.g. *Castanopsis sclerophylla* has a more conservative growth strategy compared to *Triadica sebifera*) and in terms of the extent of their intraspecific variation (e.g. *Nyssa sinensis* and *Castanea henryi* show high intraspecific variability compared to other species).

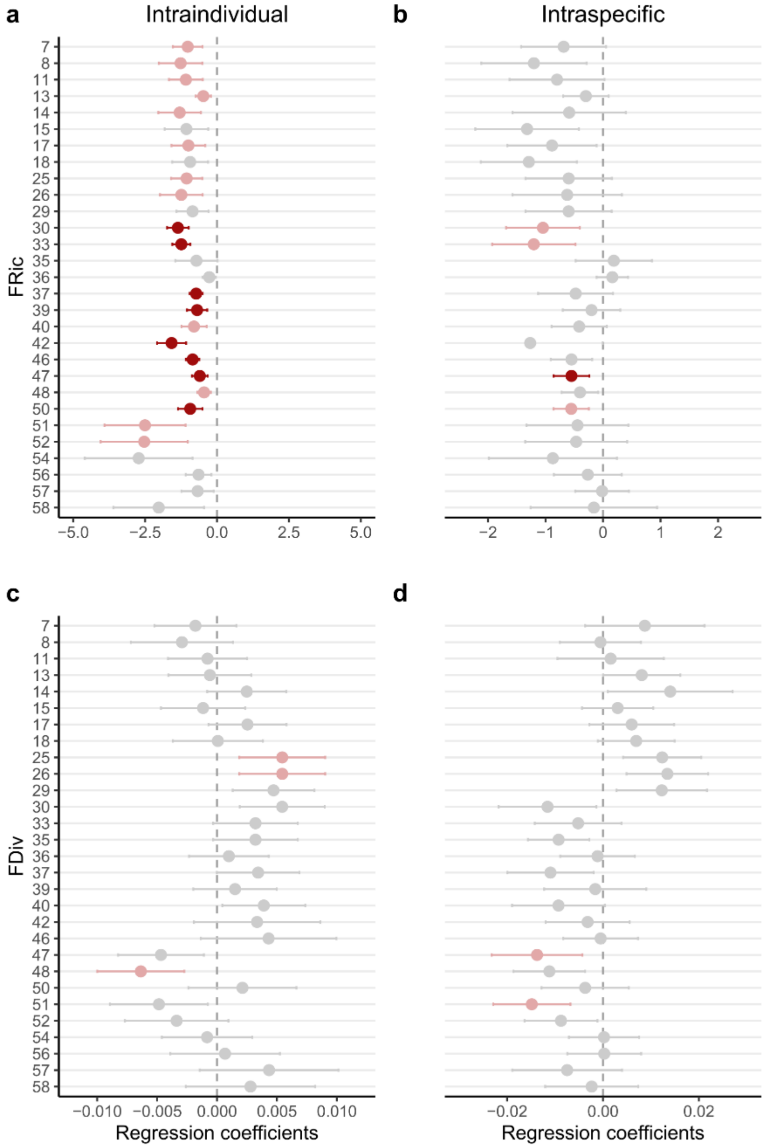

Supplementary Fig. 5. Regression coefficients for the effects of tree species richness on the intraspecific and intraindividual spectral variability on 29 principal components associated with segments of the leaf reflectance spectrum. Regression coefficients for the effects of tree species richness on the intraspecific (N = 63) and intraindividual (N = 381) variability on seven leaf functional traits and two main axes of leaf trait variability. The effects of tree species richness on intraspecific and intraindividual variability were studied for 29 principal components associated with segments of the leaf reflectance spectrum (see Fig. 3 for details about the segments). Colors represent the significance as determined by a likelihood ratio test against a model with no tree species richness effect (red $p < 0.05$, pink < 0.01, grey $p > 0.05$). Error bars correspond to the standard error.

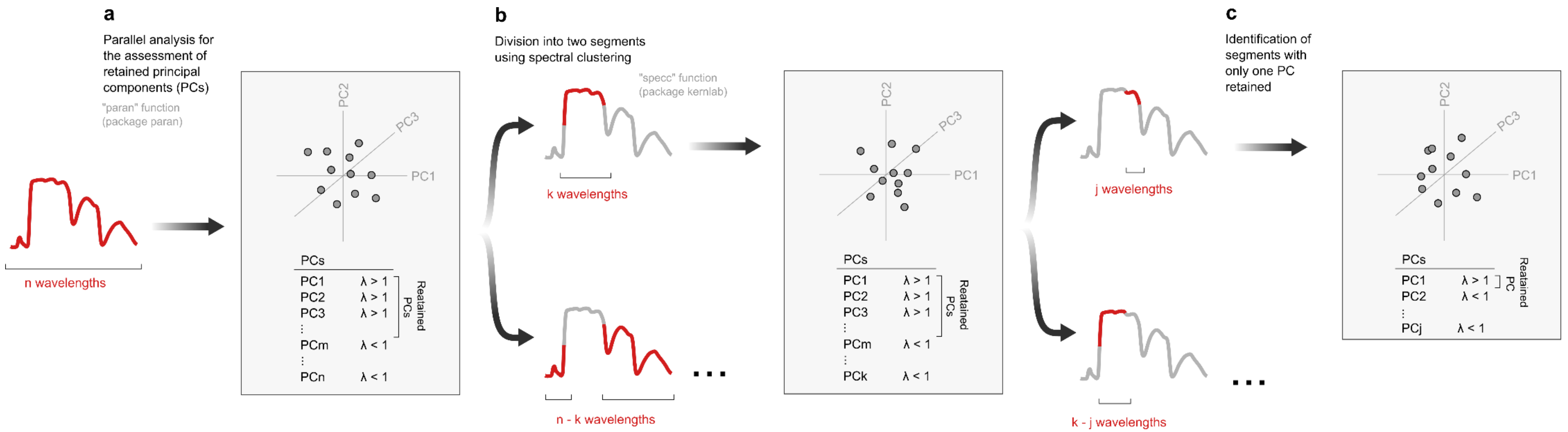

Supplementary Fig. 6. Conceptual framework for the segmentation of the leaf reflectance spectrum using the Hierarchical Spectral Clustering with Parallel Analysis (HSC-PA). (**a**) By using a Horn's parallel analysis on all the wavelengths of the leaf reflectance spectrum (2101 wavelengths), the number of retained principal components was assessed. A principal component was retained when its associated eigenvalue was higher than 1. If the number of principal components retained was higher than one, (**b**) then the wavelengths were divided into two groups (i.e. segments) using spectral clustering. The resulting segments were then used to repeat this process, always dividing the wavelengths associated to each segment into two groups until (**c**) a segment retaining one unique principal component was identified. Annotations in grey indicate the functions and the package used for the steps of the analysis. Wavelengths associated to each segment are colored in red.

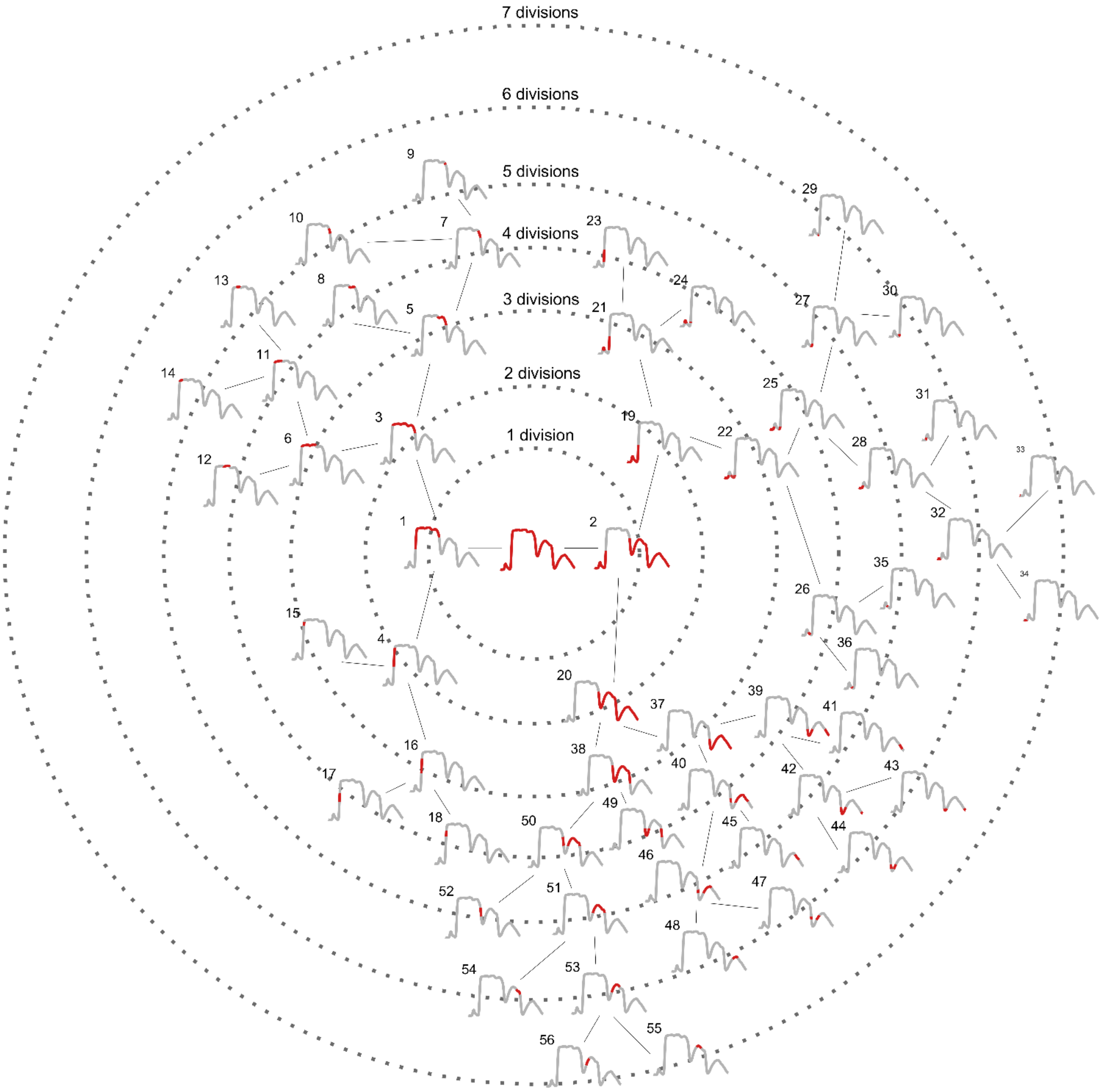

Supplementary Fig. 7. Segmentation of leaf reflectance spectrum obtained using Hierarchical Spectral Clustering with Parallel Analysis (HSC-PA). Segments are colored in red. From the center, each concentric circle represents the partition of one segment into two. Segmentation process was performed by using HSC-PA as illustrated in Supplementary Fig. 6.

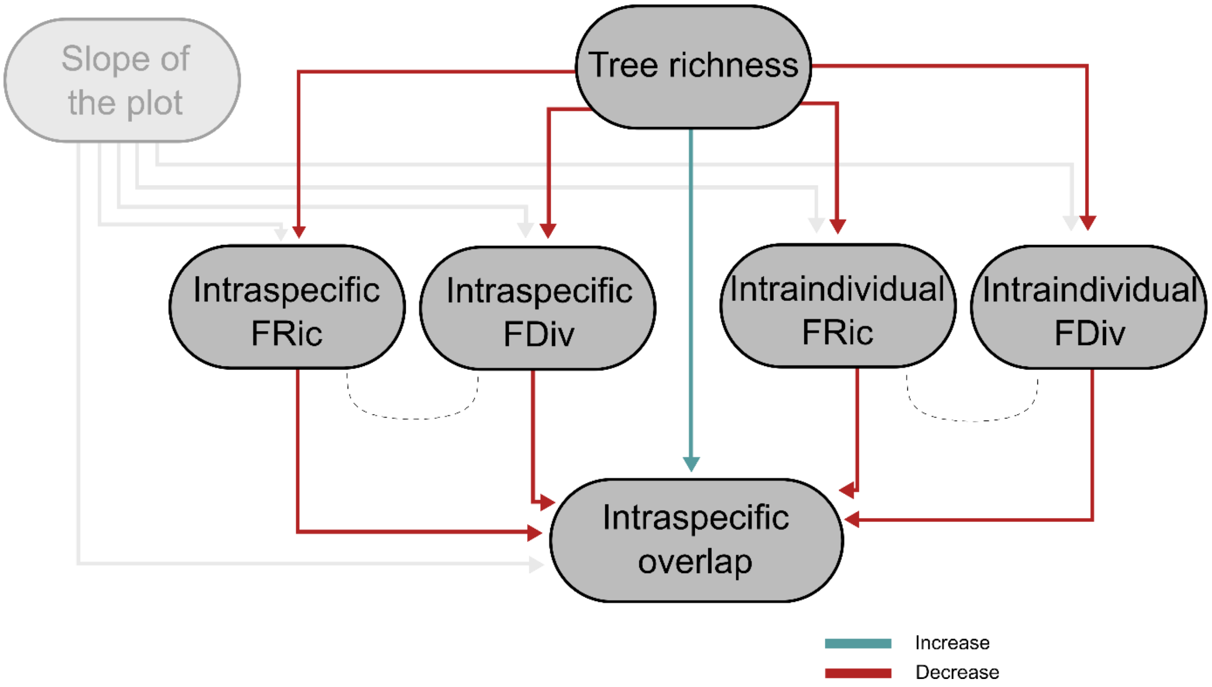

Supplementary Fig. 8. Conceptual model representing the relationships between variables that could affect intraspecific overlap in leaf functional traits. Tree species richness is expected to affect negatively the intraspecific and intraindividual trait variability (for both indices), while these are expected to have a negative effect on the intraspecific overlap. This was expected as intraindividual and intraspecific trait variability were hypothesized to act as mechanisms to guarantee complementarity in intraspecific interactions. The slope of the plot was included as a covariate in the analyses in order to control for it, but hypotheses were not formulated for its effect on the other variables. Red lines represent negative relationships, blue lines represent positive relationships and dashed lines indicate correlated error terms.

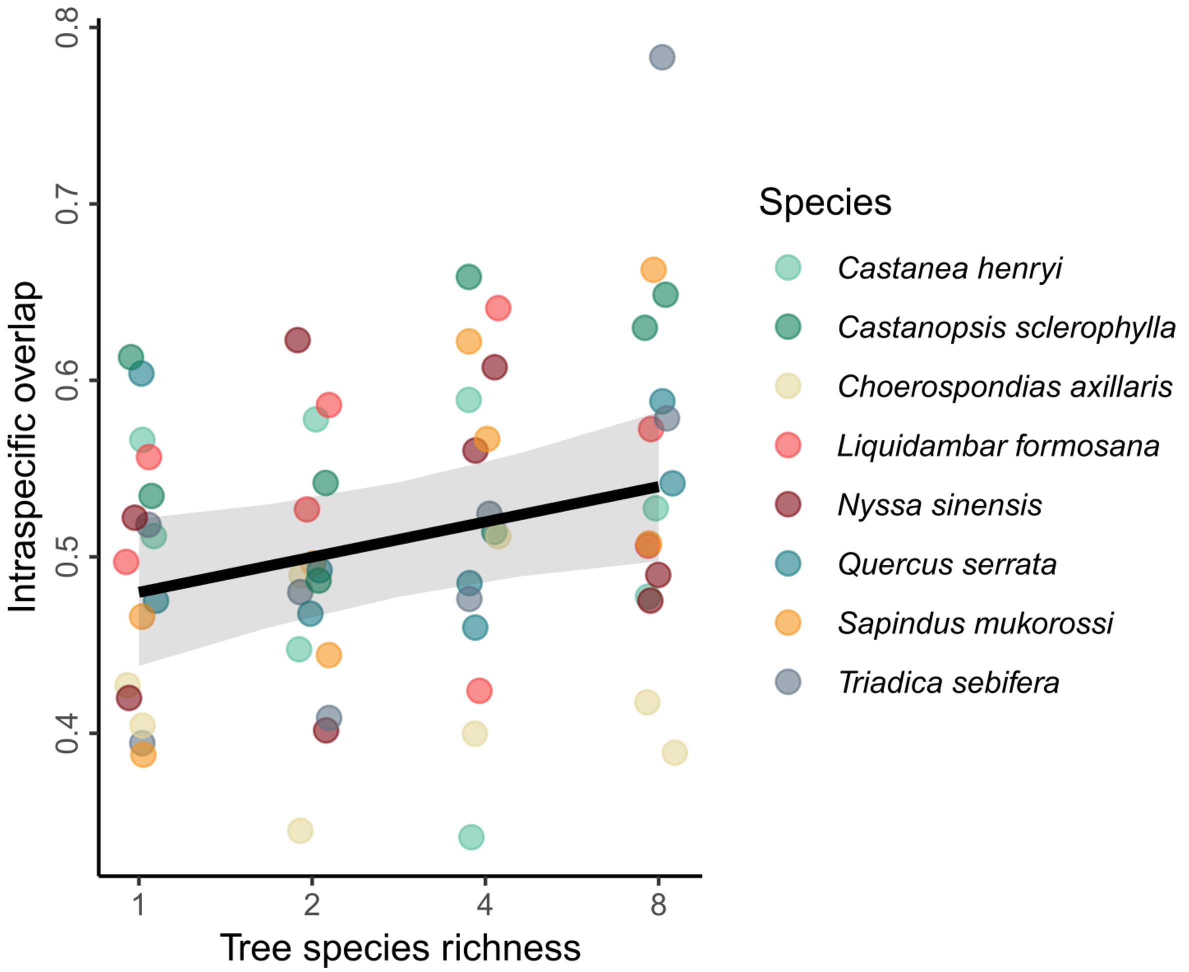

Supplementary Fig. 9. Effect of tree species richness on intraspecific overlap. The line corresponds to the results of a linear mixed-effects model that shows a significant increase of intraspecific overlap with increasing tree species richness (χ^2(df = 1) = 4.87, P = 0.03, standard estimate (β) = 0.28, N = 63). Significance was tested by using a likelihood ratio test against a model with no tree species richness effect. Grey bands represent a 95% confidence interval. Colors correspond to the different tree species included in the study, whose identity was included as a random effect in our models. The slope of the terrain was included as a covariate in the model.

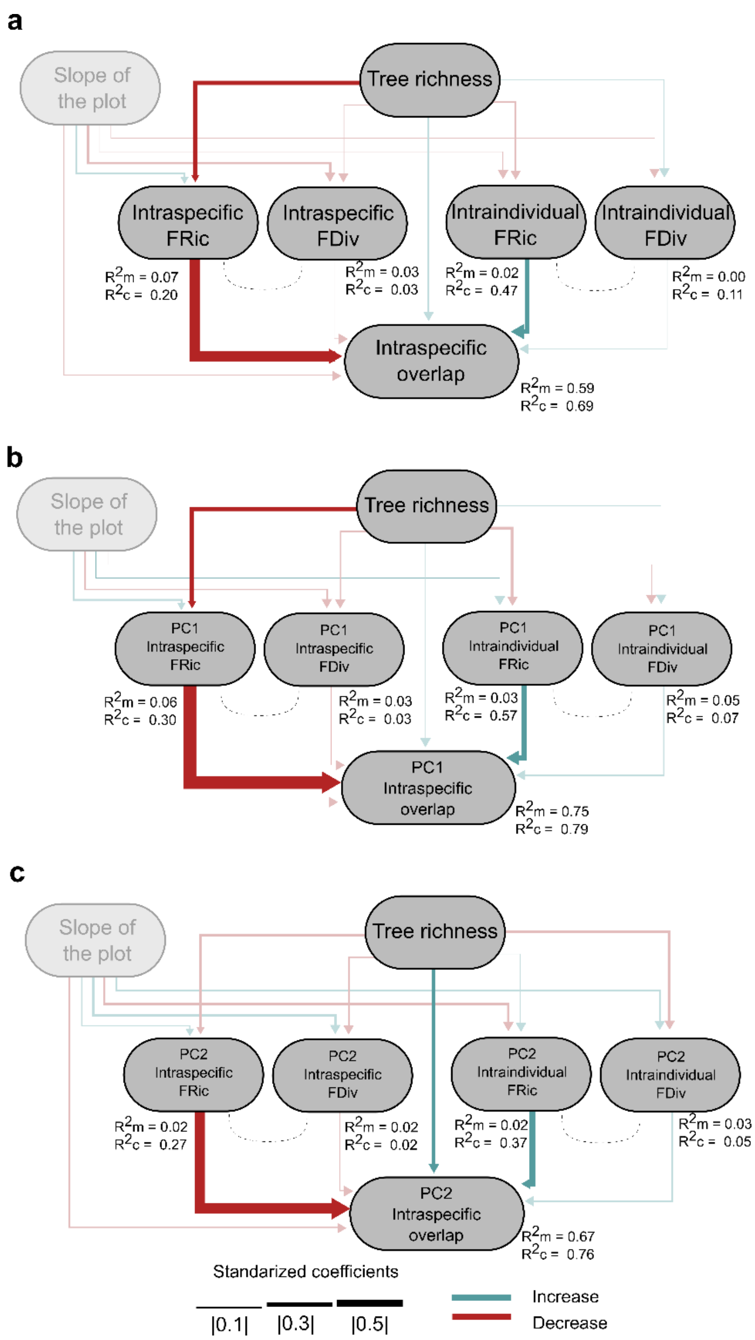

Supplementary Fig. 10. Results of non-simplified piecewise structural equation models (SEM) studying the mechanisms driving the intraspecific overlap in leaf functional traits. Results are shown for (**a**) a complete SEM based on the conceptual model defined in Supplementary Fig. 4 (Fisher's C = 6.55, df = 8, P = 0.59, N = 63), and for SEMs based on the variability on the two main axes of trait variation: (**b**) PC1 (Fisher's C = 3.24, df = 8, P = 0.92, N = 63) and (**c**) PC2 (Fisher's C = 1.92, df = 8, P = 0.98, N = 63). The width and color of the arrows indicate the strength and direction of the effects. Significant results are represented by solid lines while non-significant relationships are represented by semi-transparent lines. The marginal and conditional R^2 are indicated for every model of the piecewise SEM.

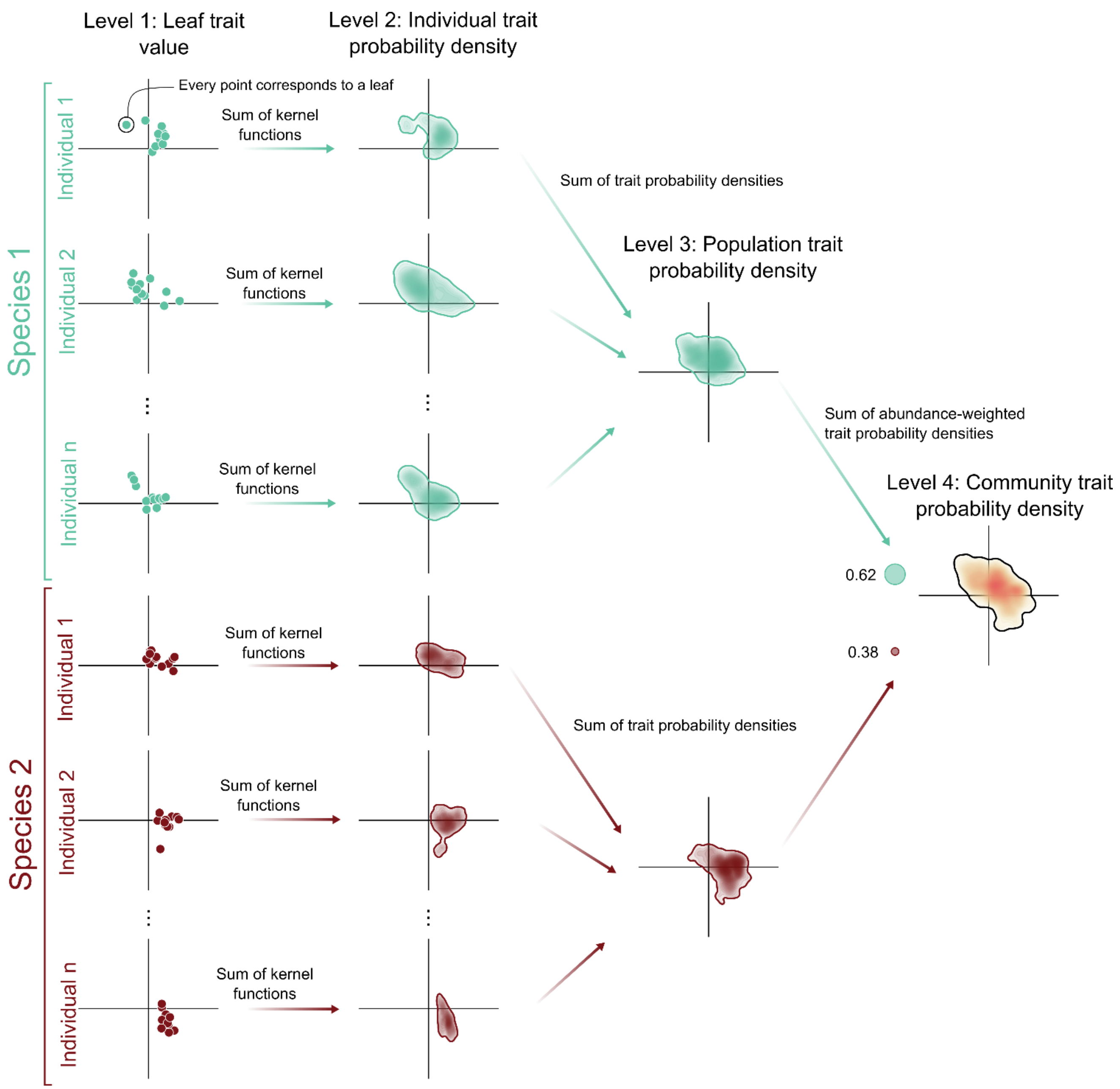

Supplementary Fig. 11. Conceptual framework for measuring community functional diversity based on individual leaf trait values (following the approach of Carmona et al.[39]). As an example, we used a community with two species (species 1 in green and species 2 in red). First (Level 1), we applied kernel density functions to the leaf trait values (represented by the leaf's position on axis 1 and axis 2 of a PCA; Fig. 3) of every tree. Next, we summarized the kernel density functions to get one trait probability density for every tree (Level 2). After this, we calculated the sum of the trait probability densities of all trees belonging to the same population to get the population trait probability density (Level 3). Finally, we aggregated the population trait probability densities (Level 4). In this step, each trait probability density was rescaled according to the relative abundance of the species in the community.

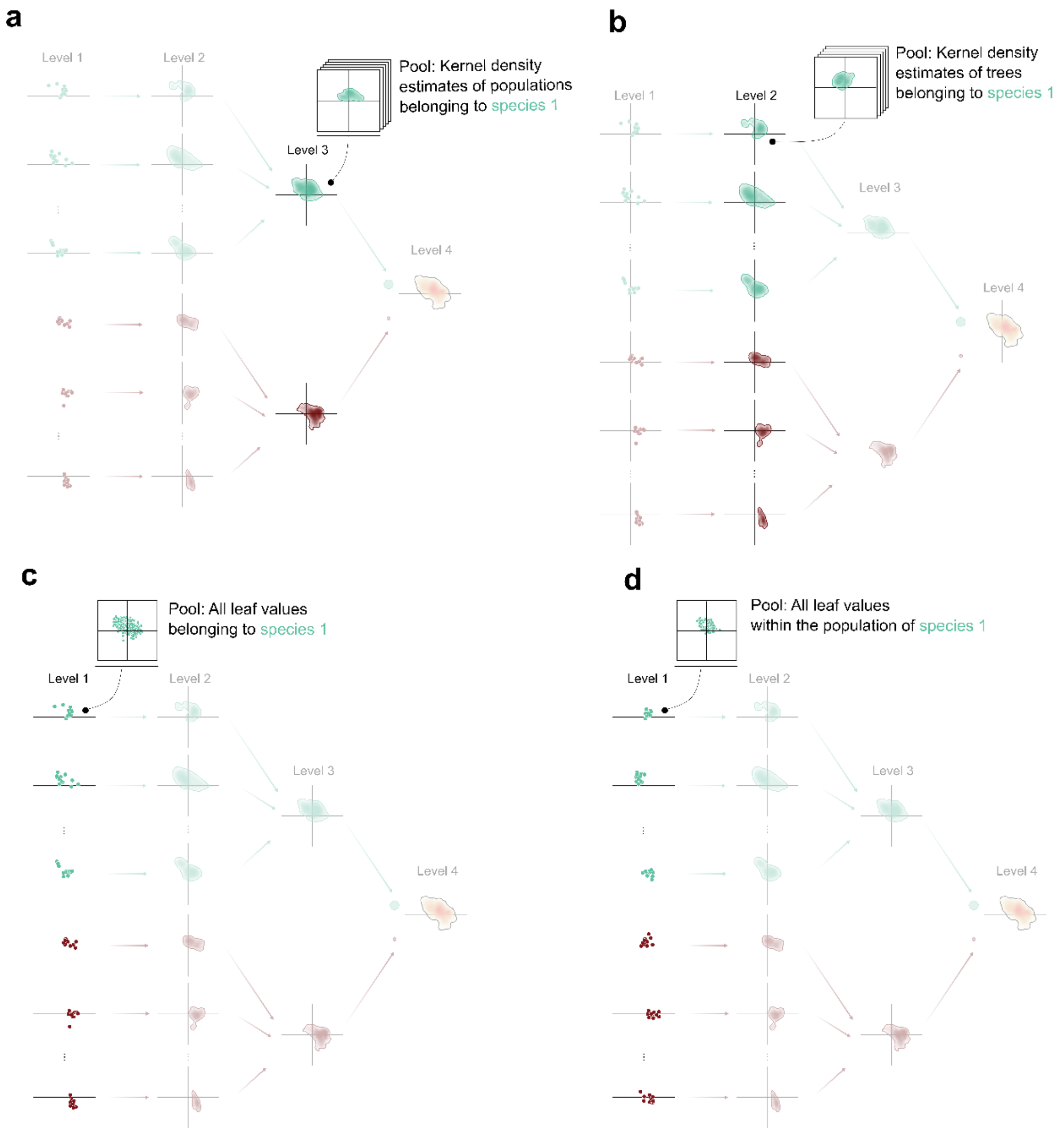

Supplementary Fig. 12. Conceptual framework for the null model approach based on the randomization of different sources of variation. Null models differ in the process for generating simulated communities by randomizing different steps of the framework for measuring functional diversity as shown in Supplementary Fig. 7. The result in every case is an assemblage with the same species composition and abundances as the observed one, but different levels of the variability occurring within the species were randomized. (**a**) The random population null model is generated by randomizing the population trait probability densities generated in step 3 by using as a pool all the different population trait probability densities calculated for that species. (**b**) The random tree null model is generated by randomizing the tree trait probability densities generated in step 2 by using as a pool all the different tree trait probability densities calculated for that species in any community. (**c**) The random leaf null model is generated by randomizing the leaves that are used to estimate the trait probability densities of trees by using as a pool all the different leaves for that species across the whole experiment. Finally, (**d**) The population-restricted random leaf null model is generated by randomizing the leaves that are used to estimate the trait probability densities of trees by using as a pool all leaves for that species within the population.

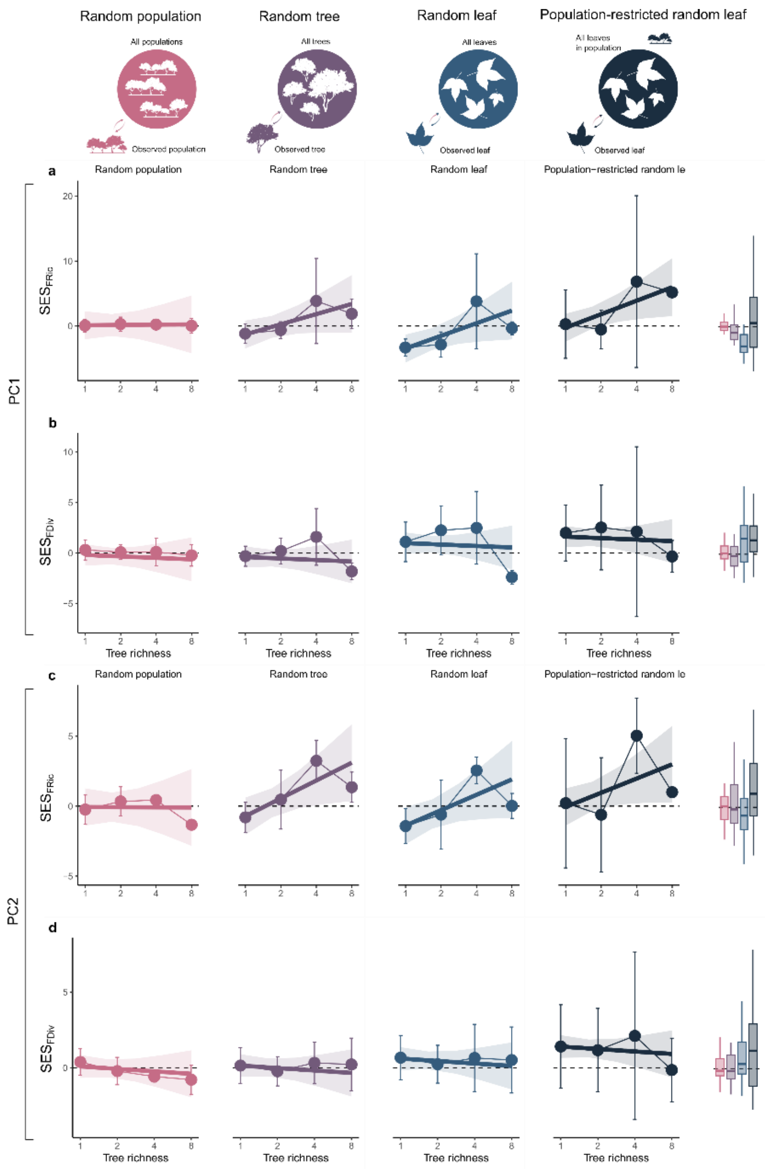

Supplementary Fig. 13. Results of linear mixed-effects models to test the joint effect of tree species richness and the type of null models on standardized effect sizes (SES) of two univariate functional indices (functional richness (FRic) and functional divergence (FDiv)) calculated from the two main axes of leaf variation (PC1 and PC2) and for four different sources of trait variation. Linear mixed-effects models show a significant effect of the interaction of tree species richness and the type of model on SES_{FRic} in the case of both axes (χ^2(df = 3) = 10.02, P = 0.02 for PC1 and χ^2(df = 3) = 8.11, P = 0.04 for PC2) and a significant effect of this, but this effect was not significant in the case of the axes of SES_{FDiv} (χ^2(df = 3) = 1.26, P = 0.74 for PC1 and P = 0.51 for PC2). However, in the case of SES_{FDiv}, there were significant effects of tree species richness (χ^2(df = 1) = 24.02, P < 0.001, regression estimates (β) = -0.05 for PC1 and χ^2(df = 1) = 32.81, P < 0.001, β = -0.08 for PC2) and the type of model (χ^2(df = 3) = 25.41, P < 0.001 for PC1 and χ^2(df = 3) = 21.41, P < 0.001 for PC2). Significance was tested by using a likelihood ratio test. Semi-transparent bands represent a 95% confidence interval. Points correspond to the mean value of SESs for each diversity level and error bars represent their standard deviation. Boxplots for comparing the values for the SESs in different models are included in the right panels. Horizontal lines inside the box indicate

the median, box limits represent the upper and lower quartiles and the whiskers are 1.5 times interquartile range.

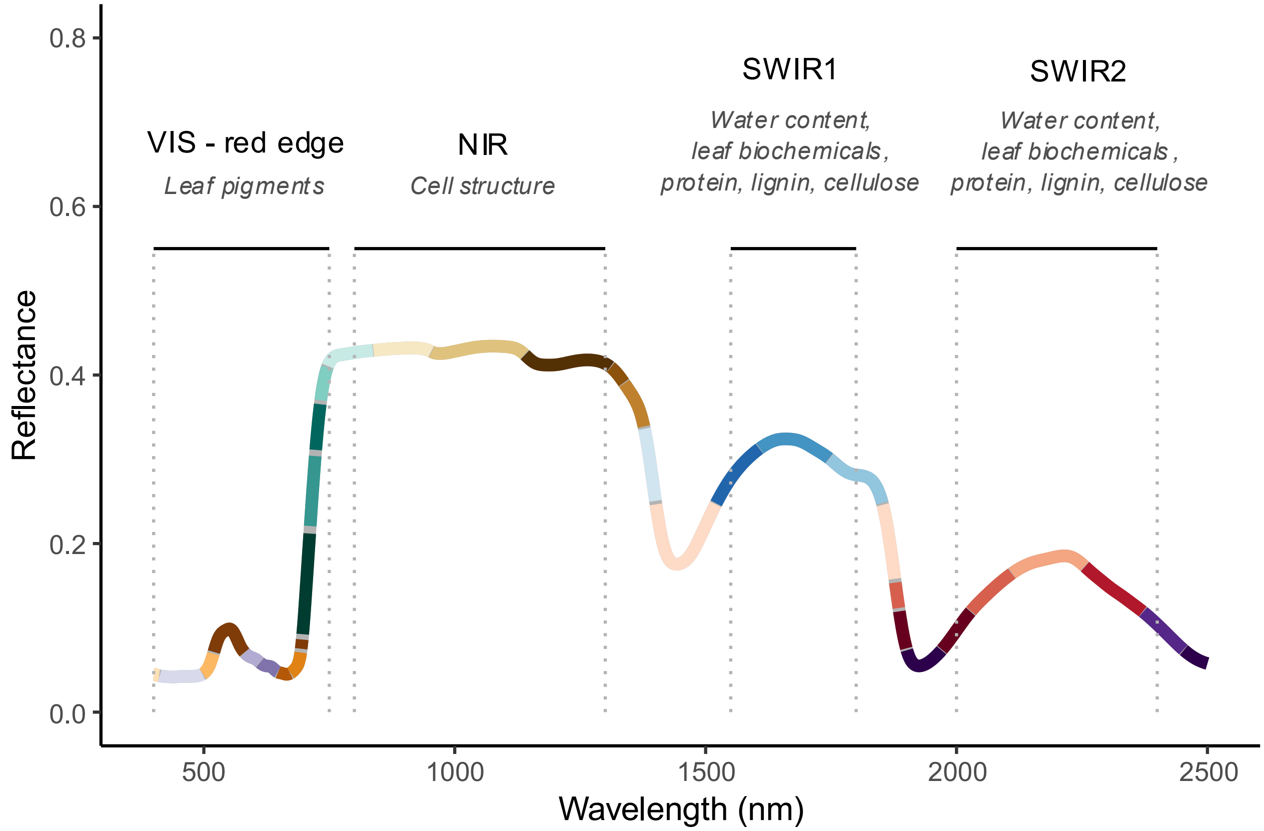

Supplementary Fig. 14. Description associations between different regions of the leaf reflectance spectrum and biochemical and structural components of leaves (adapted from Li et al.[64]). The main regions represented in the leaf reflectance spectrum include the visible range (VIS) and the red edge region (400-750 nm), the near infrared region (NIR; 800-1300 nm) and the short-wavelength infrared regions (SWIR), composed by two regions (SWIR1 and SWIR2) that are separated by water absorption bands. The line represents the mean reflectance measured at different wavelengths (from 400 to 2500nm) in our study, while colors represent the different segments illustrated in Fig. 3.

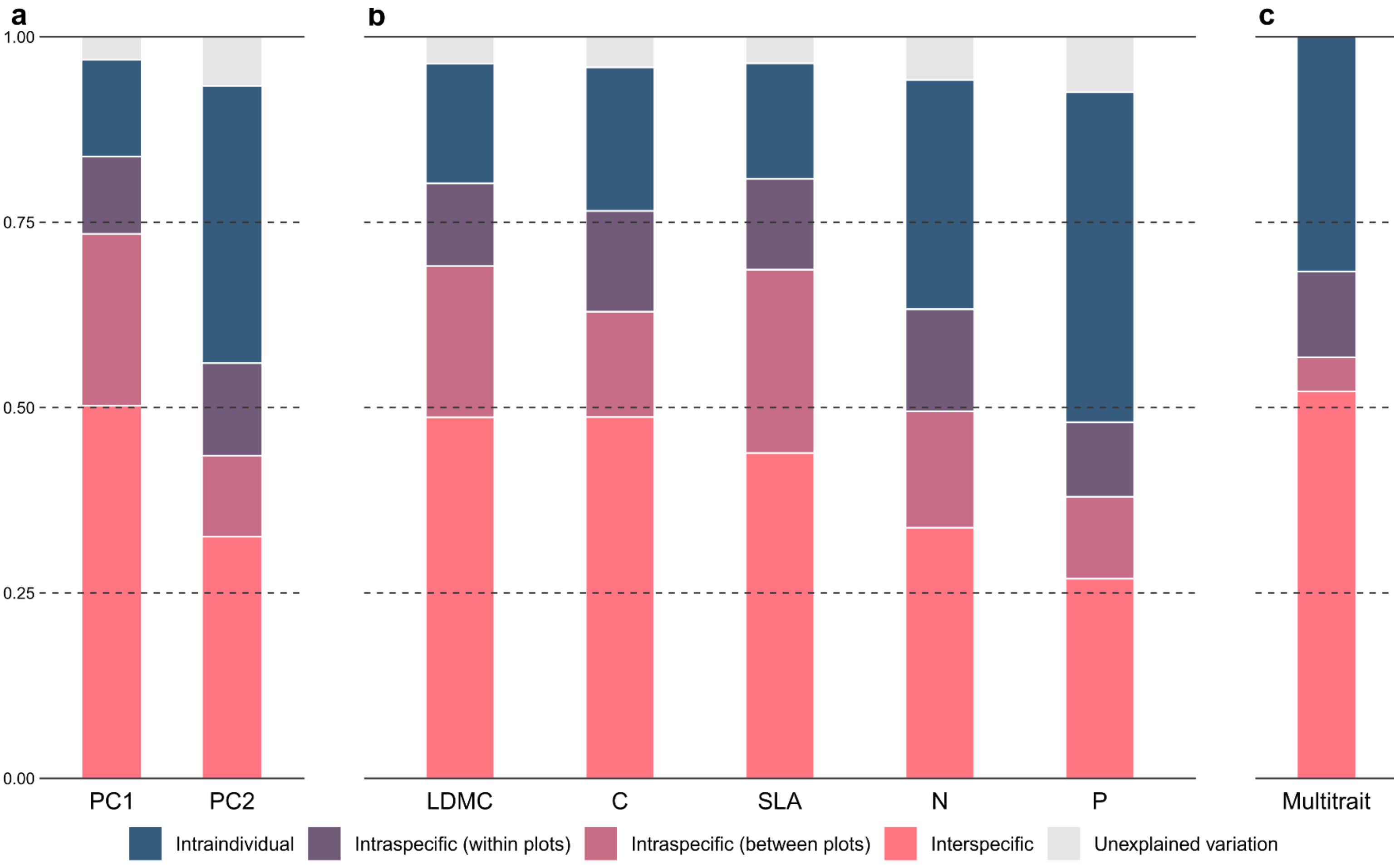

Supplementary Fig. 15. Bar plots for the variance partitioning of leaf variation. Variance partitioning was studied for (**a**) the main axes of leaf variation found in a principal component analyses (PC1 and PC2; Fig. 2A), (**b**) independently for five functional traits related to plant resource and water use (specific leaf area, SLA; leaf dry matter content, LDMC; leaf carbon content, C; leaf nitrogen content, N; leaf phosphorus content, P) and (**c**) jointly for the five leaf traits mentioned. Variance partitioning in (**a**) and (**b**) was assessed by using an intercept only linear mixed-effects model with only random effects (leaf nested in tree, in turn nested in population, in turn nested in species identity), while we used a permutational multivariate analysis of variance (PERMANOVA) with the nested structure described as a predictor for the variance partitioning in (**c**).

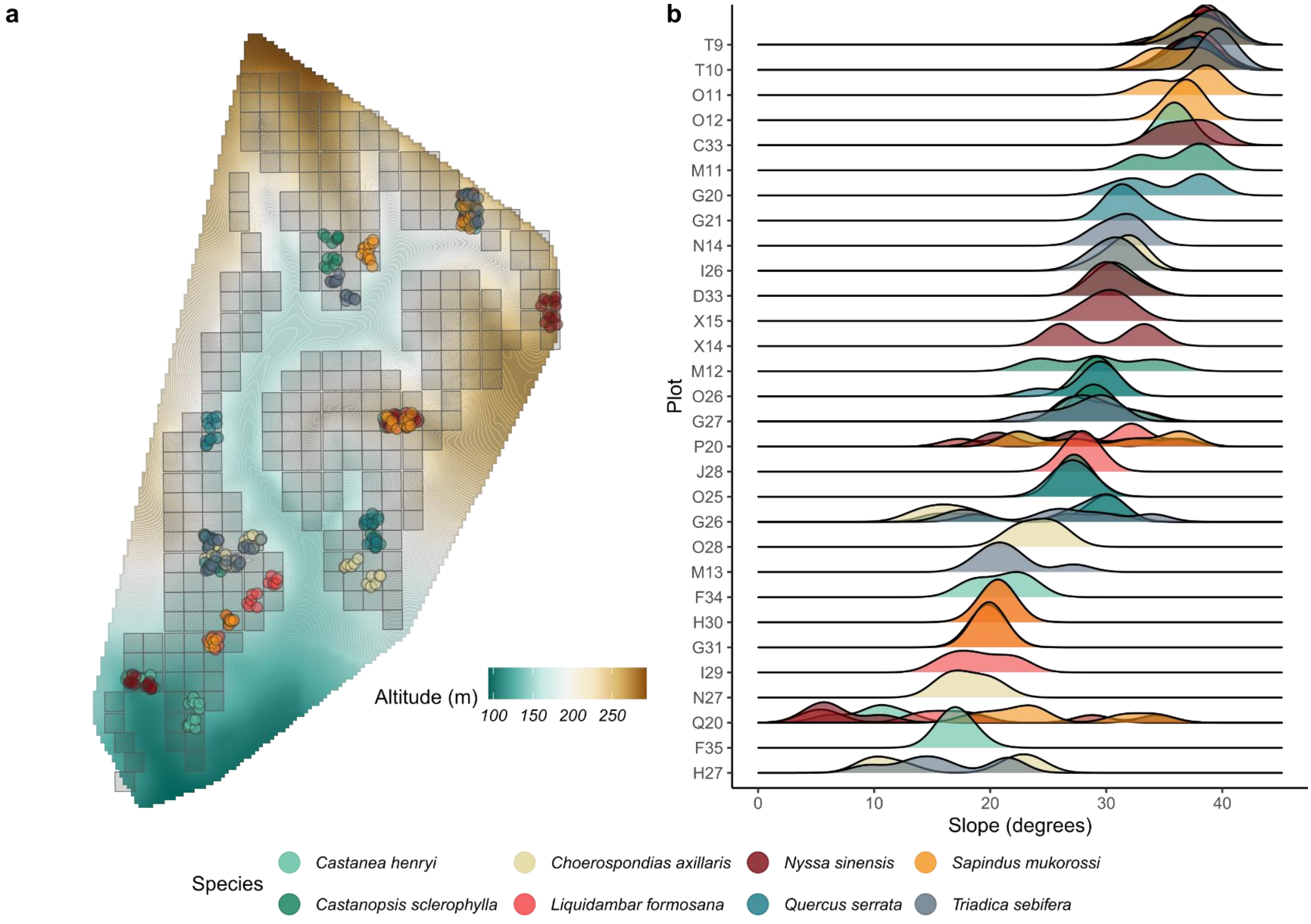

Supplementary Fig. 16. Location and slope of the sampled trees within the experimental site. (**a**) Trees were sampled across 30 plots distributed in different parts of the experiment. (**b**) Density plots of the slope of every species in every plot (based on interpolated values of the slope of the terrain obtained from a 5 m resolution digital elevation model available at https://data.botanik.uni-halle.de/bef-china/datasets/53).

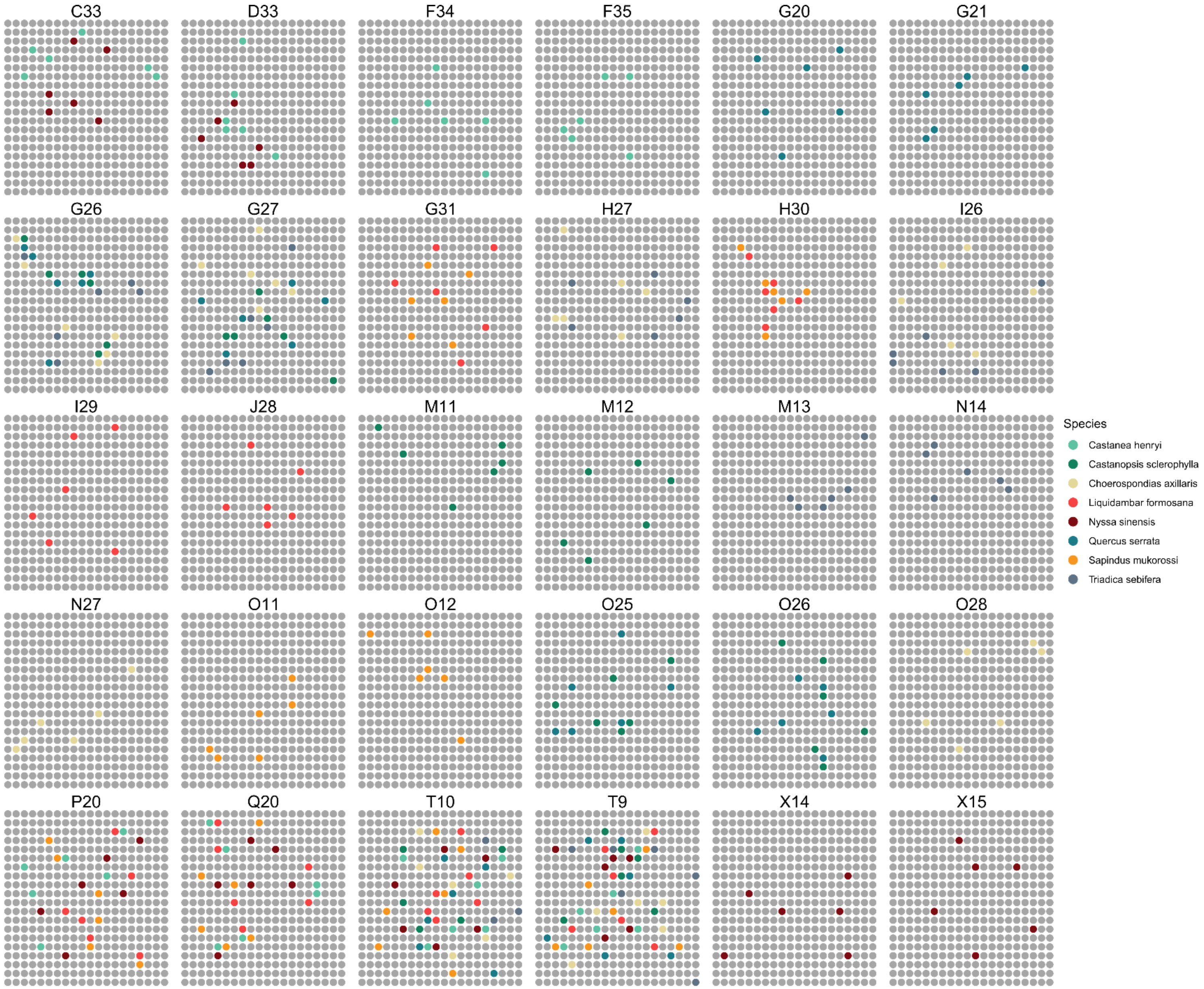

Supplementary Fig. 17. Spatial arrangement of sampled trees within each sampled plot. In each plot, 400 sampling were planted in 2009 following a grid pattern. Closest neighbors were planted at a constant distance of 1.29 m, resulting in plots of 25.8 by 25.8 m. Each planting position is represented with a circle for each plot, with grey circles indicating the position of non-sampled trees, while colored circles indicate the position of samples trees. Different species are represented in different colors.

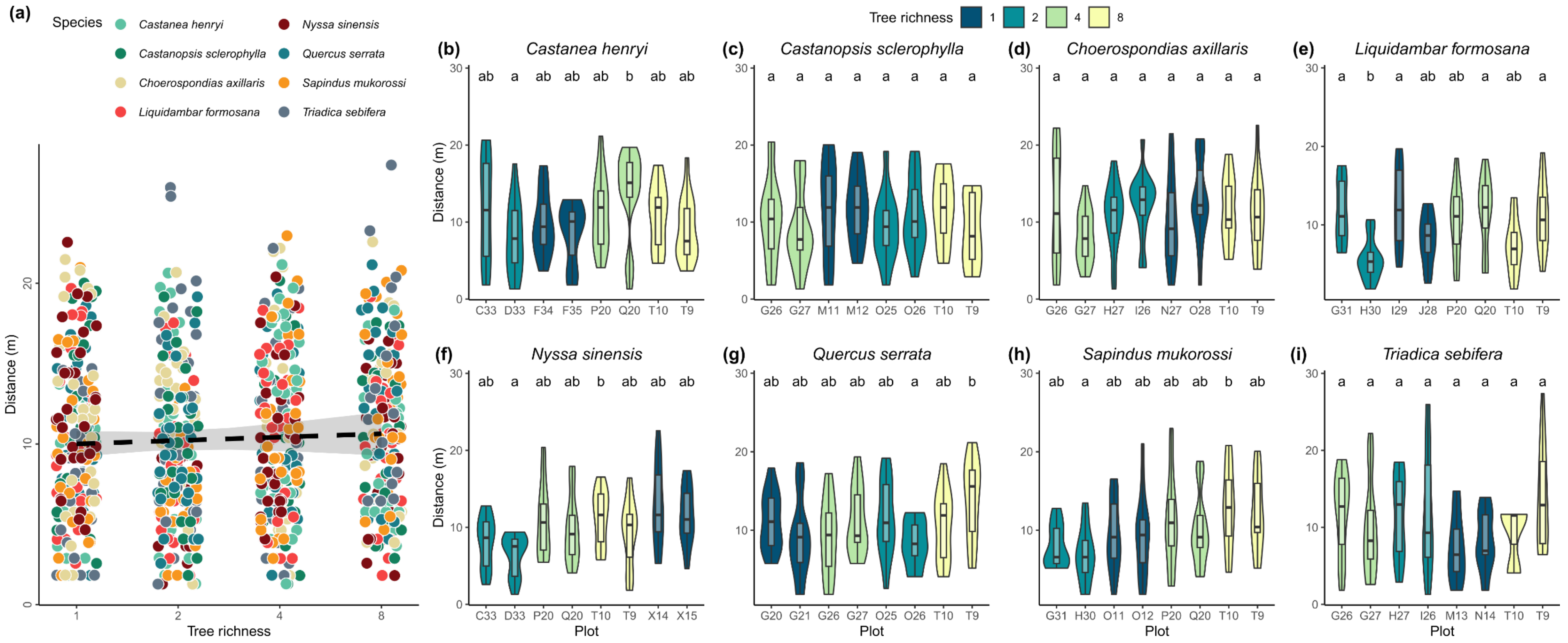

Supplementary Fig. 18. Differences in spatial distances between trees along the diversity gradient and among plots. We evaluated pairwise distances between conspecific trees along the tree species richness gradient by using a linear mixed-effects model and (**a**) results revealed no significant correlation (P = 0.46; N = 948). The linear mixed-effects model included tree richness (log2-transformed) as a predictor and plot identity and species identity as crossed random effects. Significance was tested by using a likelihood ratio test against a model with no tree species richness effect. Grey bands represent a 95% confidence interval and the shaded line indicates that the result was not significant (χ^2(df = 1) = 0.55, P = 0.46, standard estimate (β) = 0.05, N = 948). We evaluated differences in pairwise distances among plots for each species independently (b-i) and results showed specific differences in pairwise distances for some specific plots in the case of (**b**) *Castanea henryi*, (**e**) *Liquidambar formosana*, (**f**) *Nyssa sinensis*, (**g**) *Quercus serrata* and (**h**) *Sapindus mukorossi*. (post hoc two-sided t-tests). Horizontal lines inside the box indicate the median, box limits represent the upper and lower quartiles and the whiskers are 1.5 times interquartile range. The density functions around the box plots indicate the distribution of the data within each plot.

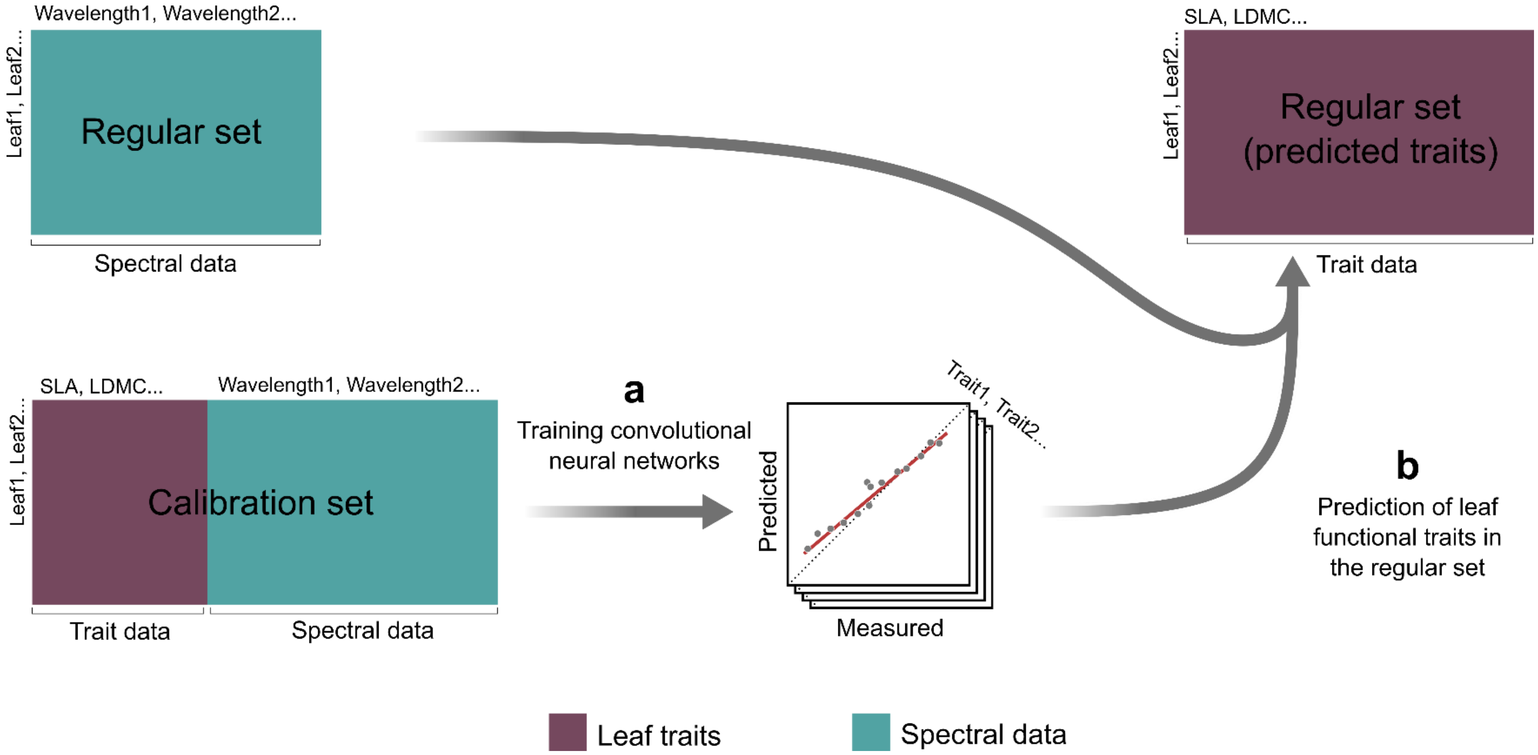

Supplementary Fig. 19. Analytical workflow used to generate a trait matrix from spectral data and the calibration sets. First, (**a**) we used a calibration set in order to train convolutional neural networks that can predict LES traits and stomatal traits, respectively, from spectral data. The calibration sets are composed of samples of the eight tree species included in this study. For each sample in these sets, leaf reflectance in the range of solar radiation (350-2500 nm) was measured. Additionally, for the samples of the calibration set, five functional traits belonging to the LES were measured: specific leaf area (SLA), leaf dry matter content (LDMC), leaf carbon content (C), leaf nitrogen content (N) and leaf phosphorus content (P). In a second step, (**b**) trained convolutional neural networks were used to predict leaf functional traits from spectral samples of the regular set. The regular set comprises leaf-level spectral samples of eight tree species collected along an experimental species richness gradient with monocultures and mixtures of 2, 4 and 8 tree species.

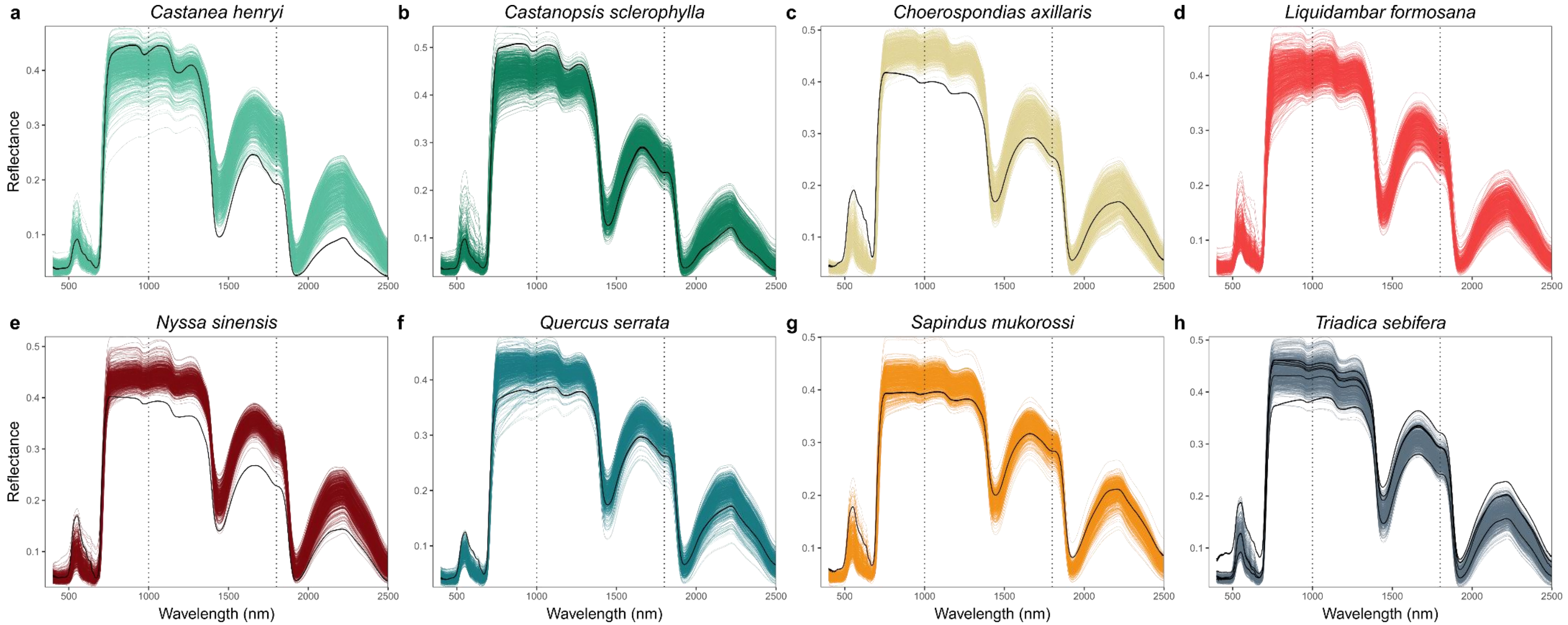

Supplementary Fig. 20. Leaf reflectance spectra for the eight study species. Spectra of all leaves collected by species (represented in different panels and different colors). Lines in black represent those spectra, which were excluded for subsequent analyses as they had a local outlier factor higher than two. Dotted vertical lines represent the limits between the sensors of the spectroradiometer use.

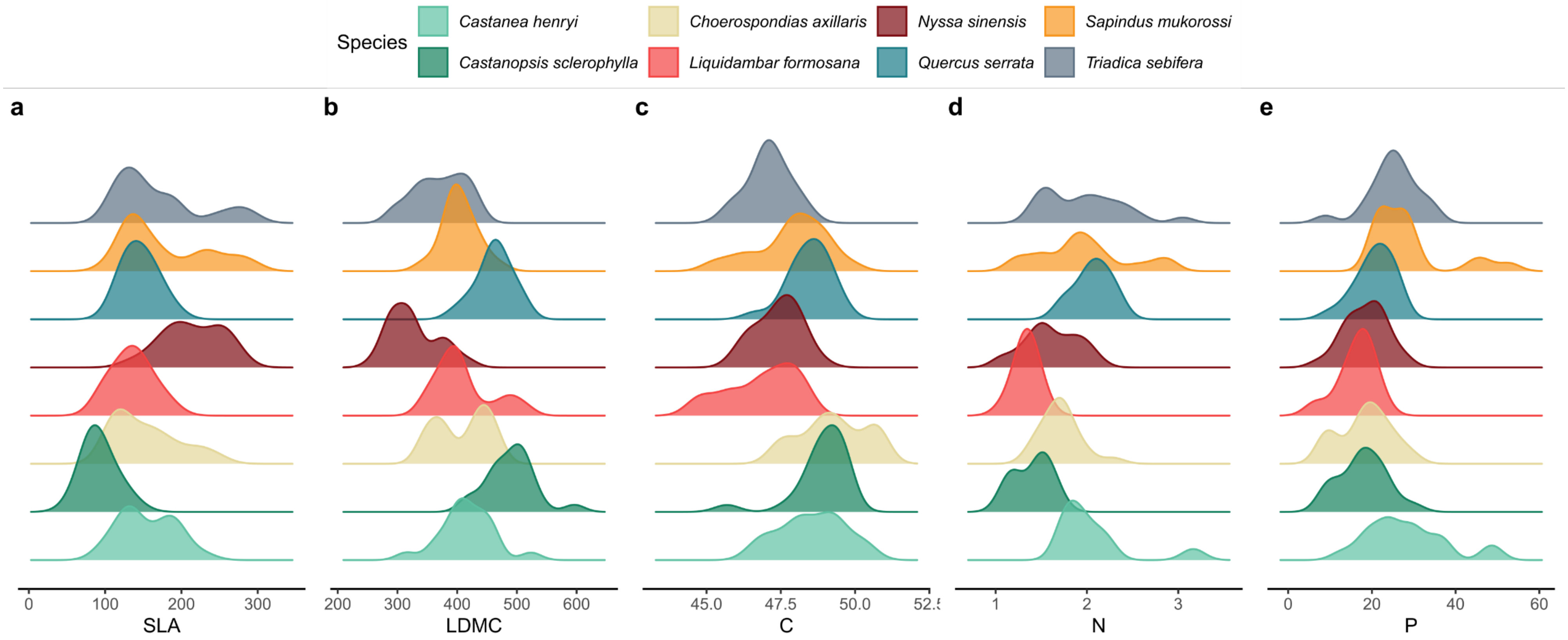

Supplementary Fig. 21. Distribution of trait values of the leaf economics spectrum for each species. Density plots are shown for (**a**) specific leaf area (SLA), (**b**) leaf dry matter content (LDMC), (**c**) leaf carbon content (C), (**d**) leaf nitrogen content, (**e**) leaf phosphorus content (P). Each row and color correspond to a species. The trait values were measured in samples collected from the field (see `Spectroscopy and laboratory analyses` section in the main text) and used to predict leaf functional traits from spectral data.

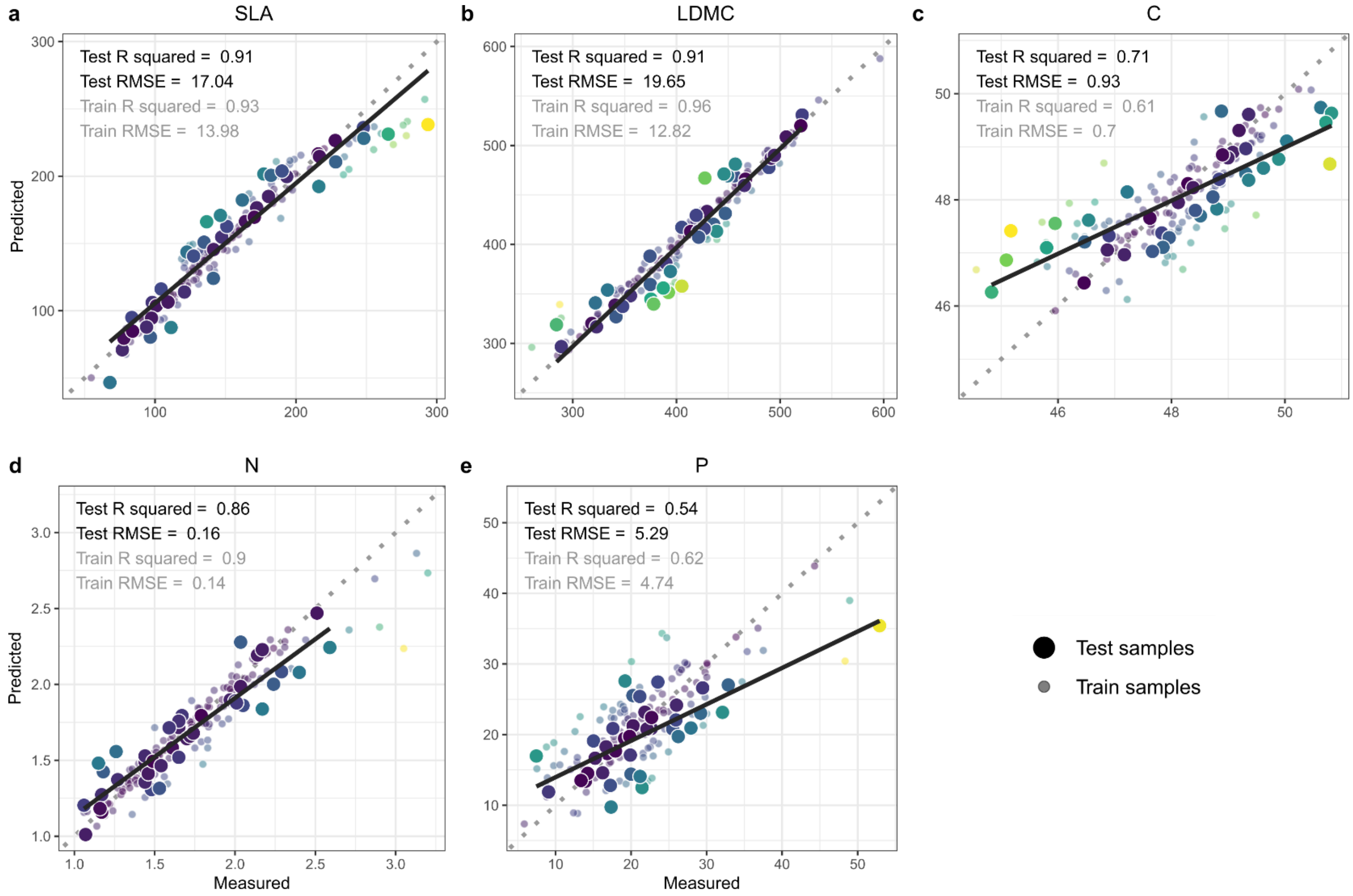

Supplementary Fig. 22. Scatter plot of predicted and measured trait values in the test and the train samples. Correlation lines correspond only to the correlation between the predicted and measured values in the test samples. The dashed grey lines indicate the optimal fit in every case. The color of the points corresponds to the distance of the value from the optimal fit (dark blue colors indicate short distance while yellowish colors correspond to higher distances).

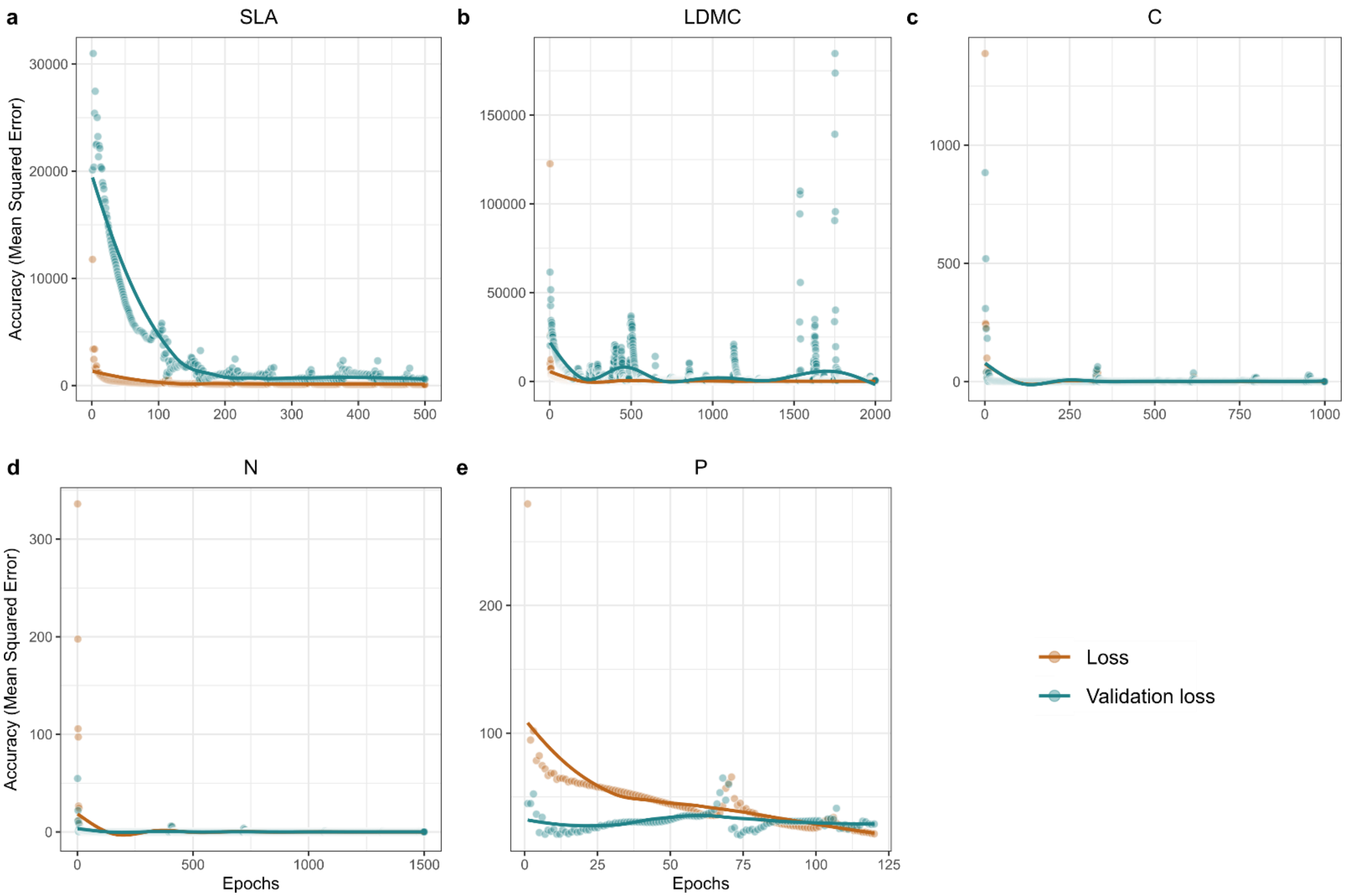

Supplementary Fig. 23. Evolution of the error during the training of convolutional neural networks for trait prediction. Changes in the mean squared error in the loss function for all samples in the training set (in brown) and for a subset of samples used for validation during the training (in blue) were registered with an increasing number of epochs during the training process of the convolutional neural networks.

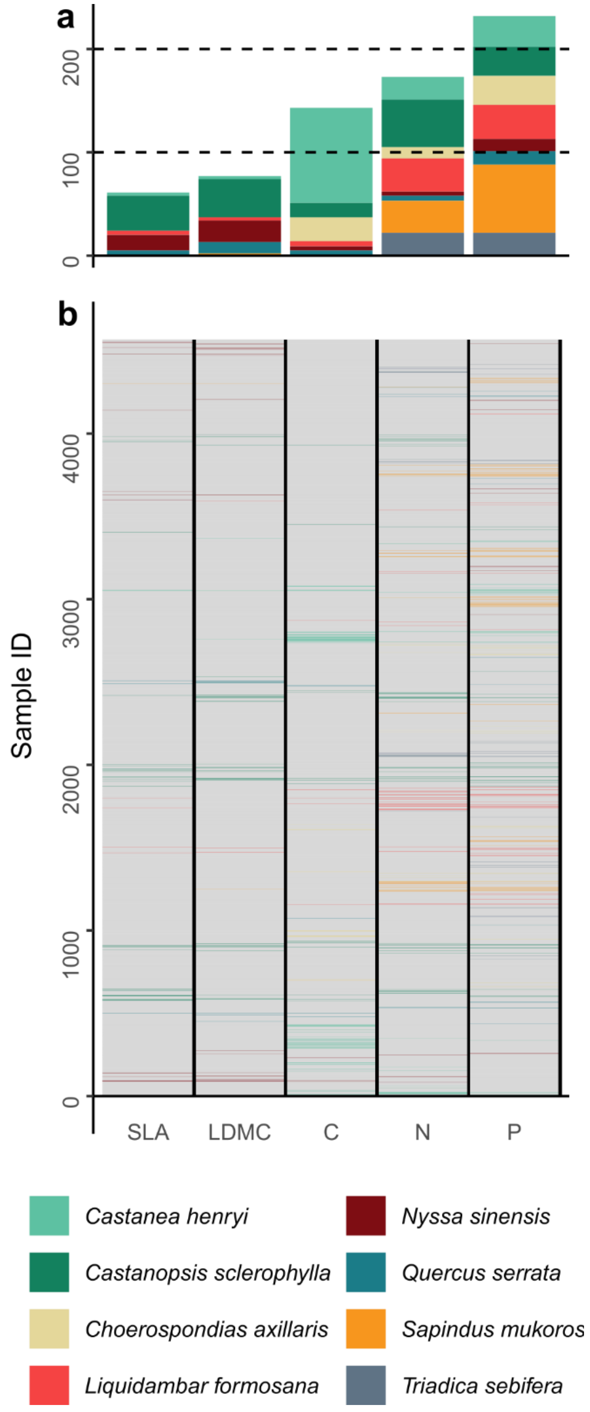

Supplementary Fig. 24. Bar plot and heatmap of the distribution of missing trait data in the leaf-level dataset. (**a**) Represents a bar plot for the number of missing values for every trait (colored by species). In (**b**), vertical colored lines represent missing values and their distribution across the dataset (as ordered in the original dataset).

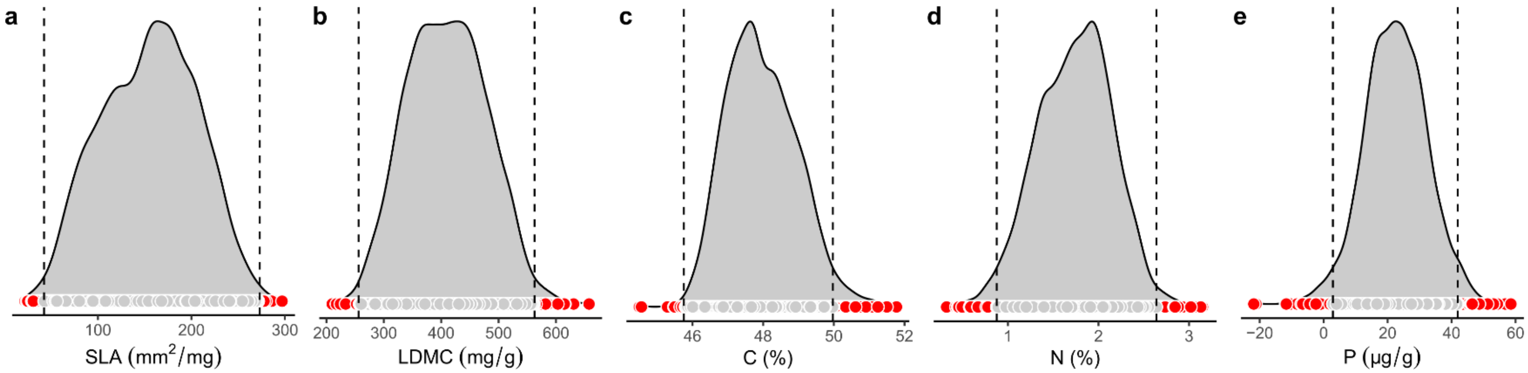

Supplementary Fig. 25. Excluded values from predicted leaf-level data for five leaf functional traits. Data excluded in trait predictions (showed in red) laid outside the interval formed by the median, plus or minus 3 median absolute deviations, as represented by the dashed vertical lines. Density plots represent the distribution of the data for each trait.

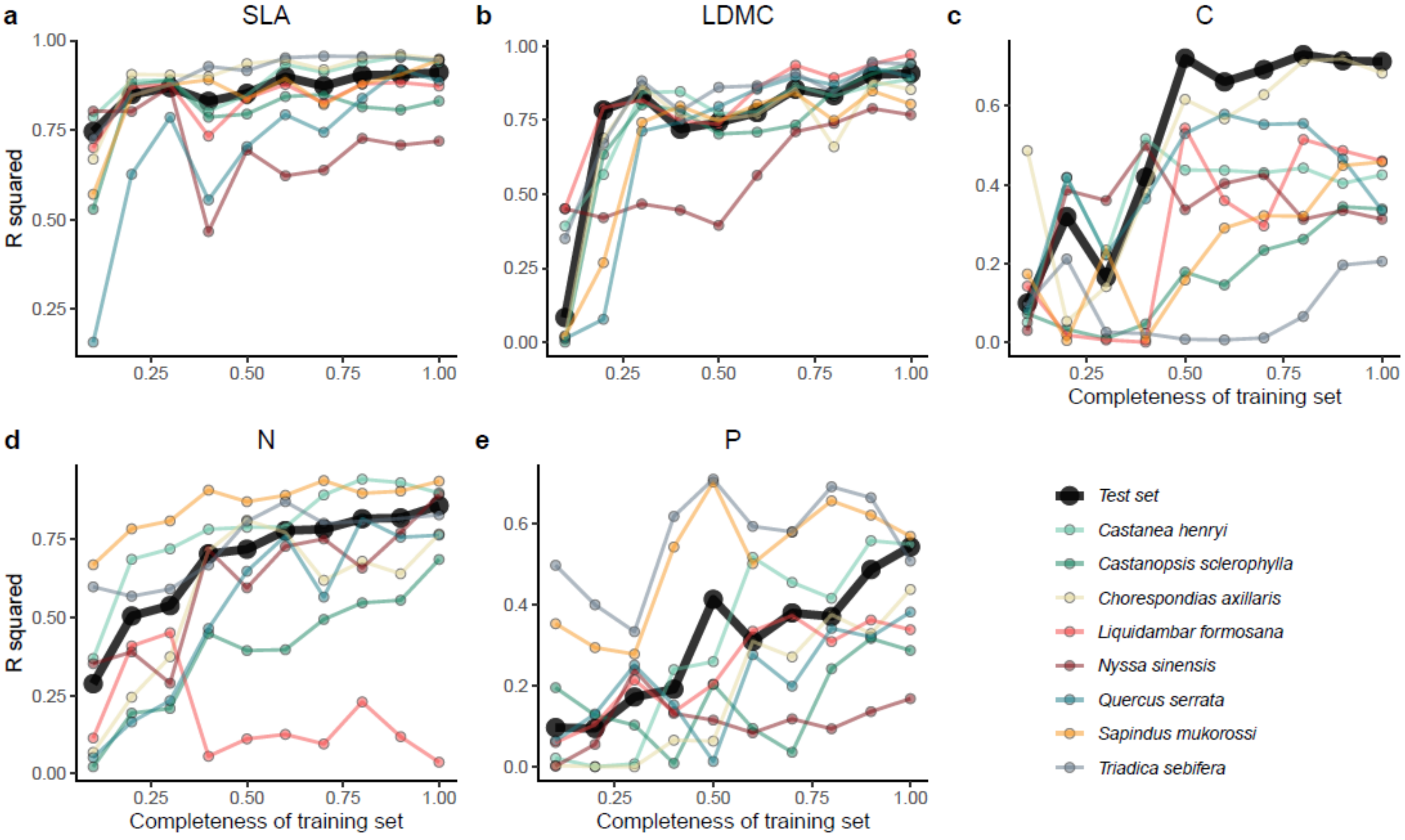

Supplementary Fig. 26. Coefficient of determination (R^2) calculated for the test set and for the samples belonging to each species independently under different scenarios of completeness of the training set. In order to evaluate if the sample size (n = 160) was sufficient to predict leaf functional traits effectively from spectral data, we simulated different scenarios of growing completeness of the training set (which was composed of the 75% of the samples of the calibration set), to evaluate changes in the predictive ability of the model in the test set and for the samples of each species independently. Therefore, we fitted predictive models using subsets of the training set representing different proportion of completeness of the training set (10%, 20%, 30%, 40%, 50%, 60%, 70%, 80%, 90% and 100% of samples), and we made sure that the number of samples per species was even in each case. Despite differences among species and traits, most of the traits, except for leaf phosphorus content (P) reached stable values of R^2 when the completeness of the training set was 0.5 (meaning that only half of the samples in the training set were used to train the model). The different pattern in P may explain the lower predictive ability for this trait.

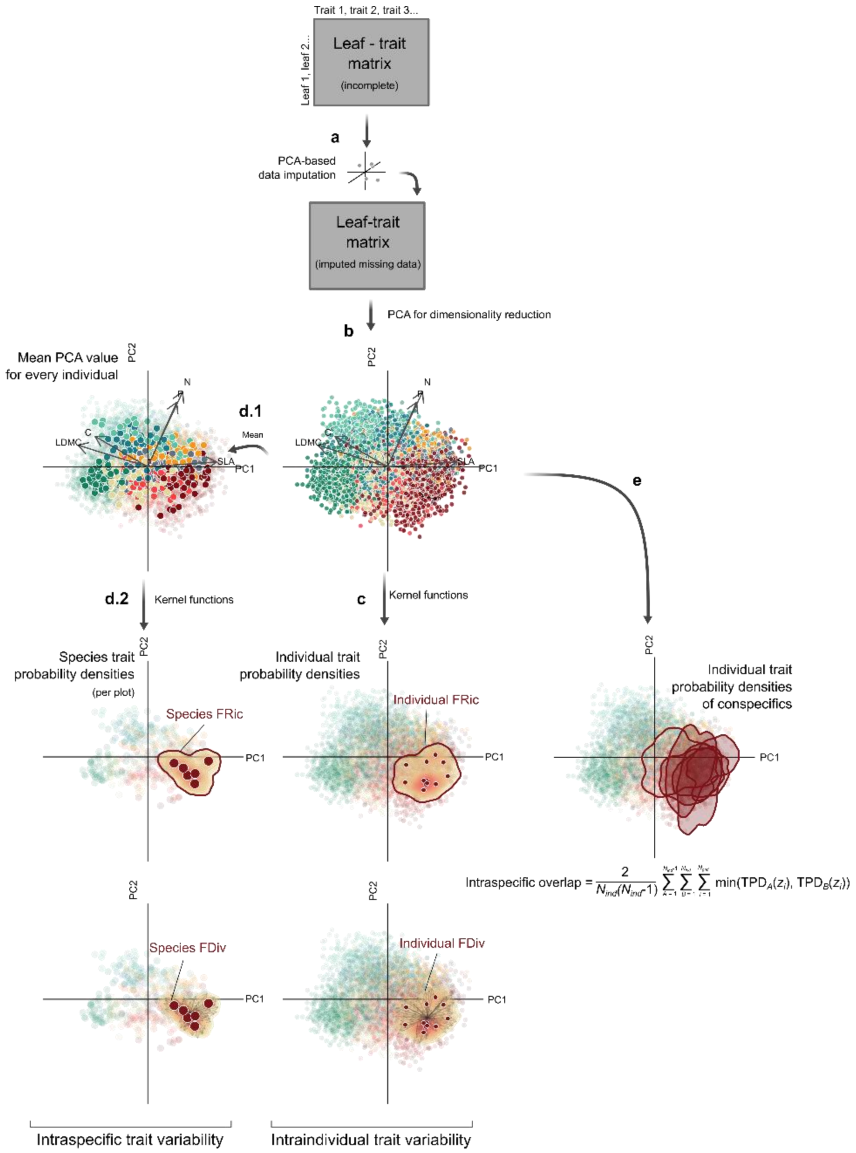

Supplementary Fig. 27. Analytical framework used to assess the metrics of intraindividual variability, intraspecific variability and intraspecific overlap. All metrics were assessed by using (**a**) the leaf-level trait matrix. Due to missing values in the matrix, a principal component analyses (PCA)-based imputation approach was used to predict the missing data from the existing ones. With the completed dataset via imputation, (**b**) we performed a PCA to reduce the dimensionality of our data and used the first two principal components which together explained almost 70% of the variation. While, (**c**) trait probability densities were estimated for individual trees from this data, (**d.1**) mean values were obtained for individual trees in order to assess (**d.2**) trait probability densities for the intraspecific trait variability. From all these trait probability densities (the ones at the individual level and the population level) functional richness and functional divergence were used to estimate intraindividual and intraspecific trait variability. Last, (**e**) the trait probability densities estimated at the individual level for conspecifics (individuals from the same species occurring in the same plot) were used to estimate the mean intraspecific overlap of a species in a plot.

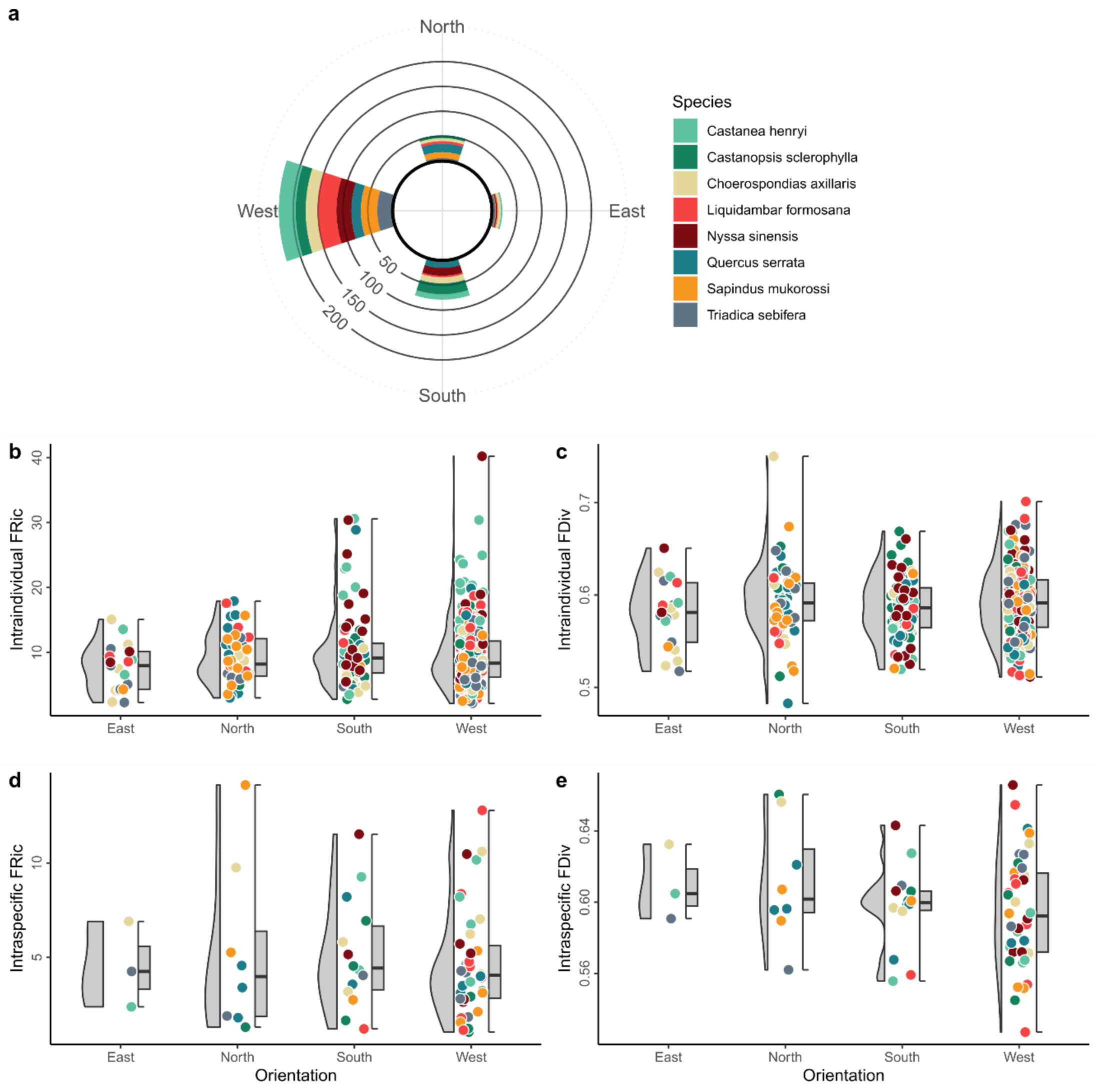

Supplementary Fig. 28. Differences in aspect among sampled trees. For each tree sampled, aspect was based on interpolated values of the slope of the terrain obtained from a 5 m resolution digital elevation model (available at https://data.botanik.uni-halle.de/bef-china/datasets/53) and it was used to assign an orientation (east, north, south, west). (a) A circular barplot indicating the number of individuals assigned to each orientation shows that the distribution of orientation is not even, but most of the sampled trees were oriented towards the west. Boxplots indicating differences on (b, d) functional richness (FRic) and (c, e) functional divergence (FDiv) were used to assess (b, c) intraindividual and (d, e) intraspecific trait variability in trees sampled for this study. No apparent differences in the mean are observed in this figure (but see Supplementary Table 8 addressing the importance of orientation as a covariate in linear mixed-effects models testing the effect of tree species richness on intraspecific and intraindividual trait variability). Horizontal lines inside the box indicate the median, box limits represent the upper and lower quartiles and the whiskers are 1.5 times interquartile range. Density plots indicate the distribution of the data within each orientation.

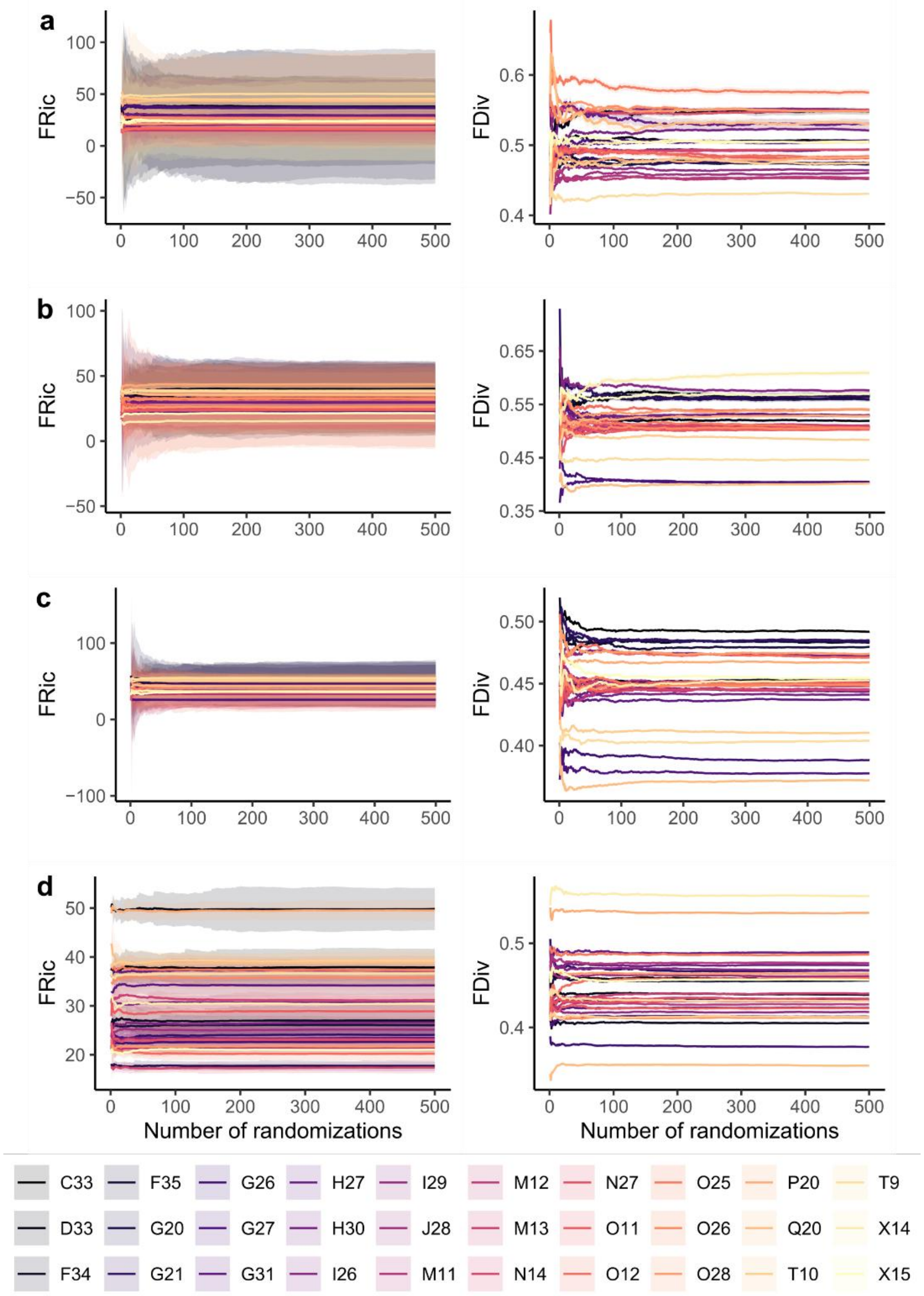

Supplementary Fig. 29. Evolution of mean and variance of simulated values of FRic and FDiv from different null models with increasing number of randomizations. In order to assess the quality of using 500 randomizations, the changes in the mean (represented by lines) and the variance (represented as dashed areas) of functional diversity indices were studied in response to the number of randomizations for the null models of (**A**) random population, (**B**) random tree, (**C**) random leaf and (**D**) population-restricted random leaf null model. Colors correspond to the different plots included in our study.

Supplementary Table 1. Summary of a principal component analysis for five leaf functional traits, including loadings, standard deviation, proportion of the variance explained by each component and the adjusted eigenvalue obtained in a Horn's parallel analysis.

Trait	PC1	PC2	PC3	PC4	PC5
SLA	0.90	-0.15	0.28	-0.15	-0.24
LDMC	-0.89	0.31	-0.13	0.19	-0.25
C	-0.67	0.50	0.45	-0.31	0.04
N	0.60	0.65	0.23	0.40	0.05
P	0.51	0.70	-0.42	-0.27	-0.01
Standard deviation	1.63	1.13	0.73	0.63	0.35
Proportion of variance	0.53	0.26	0.11	0.08	0.03
Cumulative Proportion	0.44	0.79	0.90	0.98	1.00
Adjusted eigenvalue	2.63	1.26	0.53	0.41	0.16

Supplementary Table 2. Results for linear mixed-effects models studying the effects of tree species richness on multivariate functional indices used to estimate intraspecific variability, intraindividual variability and intraspecific overlap. Standard Estimates (standard errors) and significance assessed with likelihood ratio tests are shown. The slope of the terrain (slope) was included as a covariate in the models for intraspecific variability and intraspecific overlap, while the models for intraindividual variability included slope and diameter at breast height (DBH) as covariates.

Level	Index	Tree species richness	Slope	DBH	R^2m	R^2c
Intraspecific	FRic	**-0.30(0.26), P = 0.03***	0.11(0.27), P = 0.40	-	0.07	0.20
Intraspecific	FDiv	-0.05(0.29), P = 0.70	-0.14(0.29), P = 0.31	-	0.03	0.03
Intraindividual	FRic	-0.06(0.29), P = 0.64	-0.06(0.14), P = 0.46	**0.13(0.11), P = 0.02***	0.02	0.26
Intraindividual	FDiv	-0.01(0.13), P = 0.88	-0.001(0.11), P = 0.98	0.08(0.11), P = 0.13	0.01	0.02
-	Intraspecific overlap	**0.28(0.25), P = 0.02***	-0.14(0.26), P = 0.26	-	0.06	0.28

Note: R^2m, marginal R^2; R^2c, conditional R^2; *p < 0.05.

Supplementary Table 3. Species included in the study. Species names and families from World Flora Online (https://www.worldfloraonline.org/; accessed 19 June 2024).

Species	Family
Castanea henryi Rehder & E.H.Wilson	Fagaceae
Castanopsis sclerophylla (Lindl. & Paxton) Schottky	Fagaceae
Choerospondias axillaris (Roxb.) B.L.Burtt & A.W.Hill	Anacardiaceae
Liquidambar formosana Hance	Altingiaceae
Nyssa sinensis Oliv.	Nyssaceae
Quercus serrata Murray	Fagaceae
Sapindus mukorossi Gaertn.	Sapindaceae
Triadica sebifera (L.) Small	Euphorbiaceae

Supplementary Table 4. Results for linear mixed-effects models studying the effects of tree species richness on functional indices used to estimate intraspecific variability, intraindividual variability in 30 principal components associated to segments of the leaf reflectance spectrum. Standard estimates (standard errors) and significance assessed with likelihood ratio tests are shown. Significant results ($P < 0.05$) are marked in bold. Details about the segments are shown in Fig. 3.

Segment	Intraspecific		Intraindividual	
	P value	Standard estimate	P value	Standard estimate
8	0.88	-0.02(0.27)	0.18	-0.21(0.31)
9	0.98	-0.003(0.27)	0.2	-0.17(0.28)
10	0.66	-0.06(0.27)	0.13	-0.14(0.20)
12	0.43	-0.11(0.27)	0.13	-0.26(0.35)
13	0.58	-0.07(0.27)	0.09	-0.28(0.33)
14	0.49	-0.08(0.32)	0.07	-0.29(0.32)
15	0.06	-0.25(0.28)	**0.03**	**-0.27(0.24)**
17	0.21	-0.18(0.28)	0.06	-0.16(0.16)
18	**0.04**	**-0.29(0.33)**	**0.04**	**-0.22(0.19)**
23	0.13	-0.20(0.43)	**p<0.01**	**-0.22(0.12)**
24	0.24	-0.22(0.35)	**p<0.01**	**-0.20(0.12)**
29	0.3	-0.15(0.37)	0.07	-0.17(0.19)
30	0.62	-0.07(0.42)	**0.05**	**-0.17(0.16)**
31	0.35	-0.15(0.29)	**p<0.01**	**-0.17(0.10)**
33	0.61	0.09(0.27)	0.22	-0.09(0.15)
34	0.8	0.04(0.35)	0.29	-0.09(0.18)
35	0.06	-0.29(0.31)	**p<0.001**	**-0.22(0.11)**
36	0.07	-0.26(0.25)	**p<0.01**	**-0.22(0.12)**
41	0.42	-0.10(0.24)	0.12	-0.13(0.17)
43	0.51	-0.08(0.26)	0.1	-0.15(0.17)
44	0.42	-0.10(0.25)	0.06	-0.16(0.17)
45	0.11	-0.20(0.26)	0.13	-0.13(0.17)
47	0.25	-0.15(0.25)	0.09	-0.15(0.18)
48	0.14	-0.19(0.27)	0.15	-0.13(0.18)
49	0.55	-0.08(0.27)	0.08	-0.15(0.17)
52	0.45	-0.10(0.27)	0.07	-0.14(0.15)
54	0.33	-0.13(0.27)	0.07	-0.15(0.16)
55	0.19	-0.18(0.27)	0.09	-0.16(0.18)
56	0.35	-0.13(0.27)	0.06	-0.16(0.16)

Supplementary Table 5. Results for linear mixed-effects models studying the effects of tree species richness and type of null model on standardized effect sizes of two functional indices. Significance assessed with likelihood ratio tests are shown. The interaction between the predictors is indicated by ":".

Response variable	Tree species richness	Type of null model	Tree species richness : Type of null model	R^2m	R^2c
SES_{FRic}	**P = 0.002****	**P = 0.002****	**P < 0.001*****	0.21	0.64
SES_{FDiv}	**P < 0.001*****	**P < 0.001*****	P < 0.21 ns	0.34	0.65

Note: R^2m, marginal R^2; R^2c, conditional R^2;

ns p > 0.05; **p < 0.01; ***p < 0.01.

Supplementary Table 6. Coefficient of determination (R^2) and root mean squared error (RMSE) for each of the eight species included in the study. All calibration samples per species were used to calculate R^2 and RMSE.

	SLA	LDMC	C	N	P
Species					
Castanea henryi	0.92	0.89	0.43	0.90	0.55
Nyssa sinensis	0.85	0.94	0.35	0.67	0.39
Quercus serrata	0.94	0.85	0.69	0.73	0.54
Castanopsis sclerophylla	0.89	0.97	0.54	0.33	0.44
Choerospondias axillaris	0.71	0.76	0.32	0.89	0.27
Liquidambar formosana	0.95	0.81	0.47	0.93	0.57
Sapindus mukorossi	0.95	0.94	0.23	0.83	0.51
Triadica sebifera	0.87	0.90	0.41	0.77	0.48
Castanea henryi	11.33	3.17	0.31	0.05	0.81
Nyssa sinensis	16.74	3.91	0.22	0.02	0.87
Quercus serrata	15.34	3.59	0.01	0.03	0.67
Castanopsis sclerophylla	7.85	1.79	0.77	0.03	0.66
Choerospondias axillaris	25.50	6.75	0.18	0.01	1.98
Liquidambar formosana	19.04	3.32	0.49	0.10	0.23
Sapindus mukorossi	14.08	0.61	0.51	0.06	1.38
Triadica sebifera	15.41	0.79	0.17	0.04	0.58

Supplementary Table 7. Layers and hyperparameters used for building a convolutional neural network for every trait, and coefficient of determination (R2) and root mean squared error (RMSE) for the test and the train samples.

Layer	Hyperparameter	SLA	LDMC	C	N	P
Spectral region	-	400-2500	400-2500	400-2500	1500-2400	1500-2400
1 dimension convolutional layer	Number of filters	2	2	1	2	2
1 dimension convolutional layer	Kernel size	50	2	35	77	77
Batch normalization layer	-	Yes	Yes	Yes	Yes	No
Max-pooling layer	Pool size	2	2	2	2	2
Layer flatten	-	Yes	Yes	Yes	Yes	Yes
Layer dense	Number of nodes	128	64	128	64	256
Layer dense	Number of nodes	32	16	32	16	64
Layer dense	Number of nodes	8	4	4	4	16
-	Epochs	500	2000	1000	1500	120
-	Validation Split	0.2	0.2	0.2	0.2	0.2
R^2 test	-	0.91	0.91	0.7	0.85	0.54
RMSE test	-	18.19	20.71	1.13	0.16	5.29
R^2 train	-	0.93	0.96	0.61	0.91	0.62
RMSE train	-	14.94	13.72	0.88	0.13	4.74

Supplementary Table 8. Distribution of missing trait data in the leaf-level dataset across species and traits.

	SLA	LDMC	C	N	P	Sdens
Castanea henryi	3 (0.07%)	3 (0.07%)	92 (2.01%)	22 (0.48%)	30 (0.66%)	79 (1.73%)
Castanopsis sclerophylla	34 (0.74%)	37 (0.81%)	14 (0.31%)	46 (1.01%)	28 (0.61%)	19 (0.42%)
Choerospondias axillaris	0 (0%)	0 (0%)	23 (0.5%)	11 (0.24%)	28 (0.61%)	1 (0.02%)
Liquidambar formosana	4 (0.09%)	3 (0.07%)	5 (0.11%)	32 (0.7%)	33 (0.72%)	14 (0.41%)
Nyssa sinensis	15 (0.33%)	21 (0.46%)	4 (0.09%)	4 (0.09%)	12 (0.26%)	2 (0.04)
Quercus serrata	4 (0.09%)	11 (0.24%)	5 (0.11%)	5 (0.11%)	13 (0.28%)	62 (1.36%)
Sapindus mukorossi	1 (0.02%)	2 (0.04%)	0 (0%)	31 (0.68%)	66 (1.44%)	1 (0.02%)
Triadica sebifera	0 (0%)	0 (0%)	0 (0%)	22 (0.48%)	22 (0.48%)	0 (0%)
Total	61 (1.34%)	77 (1.69%)	143 (3.13%)	173 (3.79%)	221 (4.84%)	178 (3.9%)

Supplementary Table 9. Competing models to identify the drivers of intraspecific and intraindividual trait variability. The competing models for each response variable, defined as those with a difference in Akaike information criterion (ΔAIC) relative to the simplest model (the one with the lowest AIC for each response variable) are highlighted in bold. For each model, information about the estimates of all the included explanatory variables, degrees of freedom, AIC and ΔAIC are included; '×' indicates whether orientation was included in the model. FRic, functional richness; FDiv, functional divergence; df, degrees of freedom; DBH, diameter at breast height of the tree.

Index	Level	Intercept	Tree richness	Orientation	Slope	DBH	df	AICc	ΔAICc
Fric	Intraindividual	**8.26**				**0.05**	**6**	**2161.28**	**0.00**
		**9.18**			**-0.03**	**0.05**	**7**	**2162.73**	**1.45**
		**8.51**	**-0.30**			**0.05**	**7**	**2163.18**	**1.90**
		9.25					5	2163.88	2.60
		9.39	-0.26		-0.03	0.05	8	2164.74	3.46
		9.89			-0.02		6	2165.67	4.39
		9.49	-0.29				6	2165.79	4.51
		7.58		×		0.05	9	2167.27	5.99
		10.10	-0.26		-0.02		7	2167.67	6.39
		8.57		×	-0.04	0.05	10	2168.73	7.45
		7.89	-0.31	×		0.05	10	2169.25	7.97
		8.40		×			8	2169.63	8.35
		8.87	-0.28	×	-0.04	0.05	11	2170.80	9.52
		9.11		×	-0.03		9	2171.44	10.16
		8.70	-0.29	×			9	2171.64	10.36
		9.41	-0.28	×	-0.03		10	2173.51	12.23
FDiv	Intraindividual	**0.58**				**0.00**	**6**	**-**	**0.00**
		**0.59**					**5**	**-**	**0.11**
		0.57		×			8	-	2.77
		0.57		×		0.00	9	-	2.84
		0.58	0.00			0.00	7	-	3.05
		0.58			0.00	0.00	7	-	3.08
		0.59	0.00				6	-	3.17
		0.58			0.00		6	-	3.18
		0.57	0.00	×			9	-	4.34
		0.57	0.00	×		0.00	10	-	4.35
		0.57		×	0.00	0.00	10	-	4.45
		0.57		×	0.00		9	-	4.54
		0.58	0.00		0.00	0.00	8	-	5.14
		0.58	0.00		0.00		7	-	5.24
		0.57	0.00	×	0.00	0.00	11	-	6.23
		0.58	0.00	×	0.00		10	-	6.32
FRic	Intraspecific	**5.3107969**	**-0.65**				**5**	**320.71**	**0.00**
		**4.4998872**					**4**	**322.35**	**1.64**
		**4.0613859**	**-0.79**		**0.05**		**6**	**322.44**	**1.74**
		3.4177354			0.04		5	324.71	4.00
		5.4164828		×			7	326.29	5.59
		6.1268068	-0.53	×			8	326.53	5.82
		4.9478693	-0.65	×	0.05		9	328.60	7.90
		4.7615457		×	0.02		8	329.31	8.61
FDiv	Intraspecific	**0.6033534**					**4**	**-244.60**	**0.00**
		**0.6265448**			**0.00**		**5**	**-243.95**	**0.65**
		0.6475083		×			7	-241.84	2.76
		0.6708027		×	0.00		8	-241.22	2.38
		0.6083357	0.00				5	-241.08	2.51
		0.6530627	0.00	×			8	-240.36	3.24
		0.6254638	0.00		0.00		6	-240.65	3.95
		0.6700038	0.00	×	0.00		9	-239.81	4.79
