# Supplementary material for: Intraspecific and intraindividual trait variability decrease with tree richness in a subtropical tree biodiversity experiment
Source: Nat Commun. 2025 Dec 11;16:11009. doi: 10.1038/s41467-025-67265-8 (PMC12698814; doi:10.1038/s41467-025-67265-8)
Supplement: Supplementary file 2 — Reporting Summary [file 41467_2025_67265_MOESM2_ESM.pdf]

nature portfolio

Corresponding author(s): Pablo Castro Sánchez-Bermejo

Last updated by author(s): Oct 29, 2025

Reporting Summary

Nature Portfolio wishes to improve the reproducibility of the work that we publish. This form provides structure for consistency and transparency in reporting. For further information on Nature Portfolio policies, see our Editorial Policies and the Editorial Policy Checklist.

Statistics

For all statistical analyses, confirm that the following items are present in the figure legend, table legend, main text, or Methods section.

n/a	Confirmed	
☐	☒	The exact sample size (*n*) for each experimental group/condition, given as a discrete number and unit of measurement
☐	☒	A statement on whether measurements were taken from distinct samples or whether the same sample was measured repeatedly
☐	☒	The statistical test(s) used AND whether they are one- or two-sided<br>*Only common tests should be described solely by name; describe more complex techniques in the Methods section.*
☐	☒	A description of all covariates tested
☐	☒	A description of any assumptions or corrections, such as tests of normality and adjustment for multiple comparisons
☐	☒	A full description of the statistical parameters including central tendency (e.g. means) or other basic estimates (e.g. regression coefficient) AND variation (e.g. standard deviation) or associated estimates of uncertainty (e.g. confidence intervals)
☐	☒	For null hypothesis testing, the test statistic (e.g. *F*, *t*, *r*) with confidence intervals, effect sizes, degrees of freedom and *P* value noted<br>*Give P values as exact values whenever suitable.*
☒	☐	For Bayesian analysis, information on the choice of priors and Markov chain Monte Carlo settings
☒	☐	For hierarchical and complex designs, identification of the appropriate level for tests and full reporting of outcomes
☐	☒	Estimates of effect sizes (e.g. Cohen's *d*, Pearson's *r*), indicating how they were calculated

Our web collection on statistics for biologists contains articles on many of the points above.

Software and code

Policy information about availability of computer code

Data collection	Codes used in this study are available at the Zenodo repository with the identifier https://zenodo.org/records/14190700.
Data analysis	All statistical analyses were conducted in the R environment with R version 4.2.3 and Python version 3.11.0. Details about dependencies are available at the Zenodo repository: https://zenodo.org/records/14190700.

For manuscripts utilizing custom algorithms or software that are central to the research but not yet described in published literature, software must be made available to editors and reviewers. We strongly encourage code deposition in a community repository (e.g. GitHub). See the Nature Portfolio guidelines for submitting code & software for further information.

Data

Policy information about availability of data

All manuscripts must include a data availability statement. This statement should provide the following information, where applicable:

- Accession codes, unique identifiers, or web links for publicly available datasets
- A description of any restrictions on data availability
- For clinical datasets or third party data, please ensure that the statement adheres to our policy

The data used in this study are available at the Zenodo repository with the identifier https://doi.org/10.5281/zenodo.14190699.

Research involving human participants, their data, or biological material

Policy information about studies with human participants or human data. See also policy information about sex, gender (identity/presentation), and sexual orientation and race, ethnicity and racism.

Reporting on sex and gender	N/A
Reporting on race, ethnicity, or other socially relevant groupings	N/A
Population characteristics	N/A
Recruitment	N/A
Ethics oversight	N/A

Note that full information on the approval of the study protocol must also be provided in the manuscript.

Field-specific reporting

Please select the one below that is the best fit for your research. If you are not sure, read the appropriate sections before making your selection.

☐ Life sciences ☐ Behavioural & social sciences ☒ Ecological, evolutionary & environmental sciences

For a reference copy of the document with all sections, see nature.com/documents/nr-reporting-summary-flat.pdf

Ecological, evolutionary & environmental sciences study design

All studies must disclose on these points even when the disclosure is negative.

Study description	Along an experimental diversity gradient with mixtures of one, two, four and eight tree species, we sampled 4,568 leaves from 381 trees belonging to eight different species to study changes in intraspecific and intraindividual leaf trait variability and assessed their contribution to community functional diversity. In the experiment, the trees are arranged according to a 'broken-stick' design, which involves dividing the species pool into two equal groups for each subordinate richness level. Therefore, we sampled 15 different tree compositions (eight monocultures, four two-species mixtures, two four-species mixture and one eight-species mixtures) and every tree composition was replicated twice, resulting in 30 plots. In every plot, six individuals were sampled per species and 12 leaves were collected per individual.
Research sample	The sampled included 4,568 leaves from 384 trees belonging to eight species typical from subtropical forests in the region: Castanea henryi Rehder & E.H. Wilson, Castanopsis sclerophylla (Lindl. & Paxton) Schottky, Choerospondias axillaris (Roxb.) B.L.Burtt & A.W.Hill, Liquidambar formosana Hance, Nyssa sinensis Oliv., Quercus serrata Murray, Sapindus mukorossi Gaertn. and Triadica sebifera (L.) Small.
Sampling strategy	The sample size represented a trade-off between the number of leaves to represent different parts of the tree crown and the number of individuals to assess intraspecific trait variability in a population. Thus, the dataset aimed to represent both intraspecific and intraindividual trait variability while allowing enough statistical power for the analyses
Data collection	For every tree, Pablo Castro Sánchez-Bermejo collected 12 fully developed leaves free from apparent mechanical or pathogen damage at three different heights and four different orientations of the crown. Immediately after collection, leaves were stored in sealable plastic bags with moistened tissue. Samples were transported in an isothermal bag equipped with cooling bags to prevent dehydration. In the laboratory, samples were temporarily stored at 6–8 °C for a maximum of 12 hours before further processing.
Timing and spatial scale	Sampling took place from mid-August to mid-September 2023. This period was chosen because during this period all sampled tree species had already produced leaves, but leaf senescence had not started yet. Regarding the spatial scale, all plots had the same size (25.8 x 25.8 m). In order to avoid possible effects of leaf phenology or plot location within the experiment, visits to the plots were randomized.
Data exclusions	The theoretical number of 384 trees to sample was reduced to 381 due to the high mortality of Triadica sebifera in one of the plots (three theoretical individuals could not be sampled). As a result, for the analyses of the variability at the population level, the populations of Triadica sebifera with three missing individuals was excluded, resulting in a total of 63 populations instead of the 64 planned. In addition, 25 leaf spectra were considered as outliers and excluded from the analyses as they had a value higher than two for the local outlier factor.
Reproducibility	Location of every individual tree within the experiment and the plot is known and documented in our dataset. In addition, all protocols for the acquisition of samples and sampling processing in the lab are documented. Last, data and R codes to reproduce the analyses are available at the Zenodo repository: https://zenodo.org/records/14190700.
Randomization	The selection of trees within every plot, was always random. The sampling of leaves attempted to represent different orientations and height of the canopy. However, within each height and orientation, leaves were selected randomly.
Blinding	Blinding is not possible for our study because we collected data from fieldwork.

Did the study involve field work? [x] Yes [ ] No

Field work, collection and transport

Field conditions	The climate in the field site is subtropical with a mean annual temperature of 16.5°C (ranging from 0.4°C in January to 34.2°C in July) and mean annual precipitation of 1,821 mm. During our sampling campaign in mid-August to mid-september 2023, mean temperatures ranged between 25 and 30ºC
Location	The BEF-China experiment is located in Xingangshan, in Jiangxi Province, China (lat. 29°08′11″N, long. 117°90′93″E).
Access & import/export	BEF-China was established with permission of the authorities and access to the experiment did not require any permit.
Disturbance	N/A

Reporting for specific materials, systems and methods

We require information from authors about some types of materials, experimental systems and methods used in many studies. Here, indicate whether each material, system or method listed is relevant to your study. If you are not sure if a list item applies to your research, read the appropriate section before selecting a response.

Materials & experimental systems

n/a	Involved in the study	
[x]	[ ]	Antibodies
[x]	[ ]	Eukaryotic cell lines
[x]	[ ]	Palaeontology and archaeology
[x]	[ ]	Animals and other organisms
[x]	[ ]	Clinical data
[x]	[ ]	Dual use research of concern
[ ]	[x]	Plants

Methods

n/a	Involved in the study	
[x]	[ ]	ChIP-seq
[x]	[ ]	Flow cytometry
[x]	[ ]	MRI-based neuroimaging

Dual use research of concern

Policy information about dual use research of concern

Hazards

Could the accidental, deliberate or reckless misuse of agents or technologies generated in the work, or the application of information presented in the manuscript, pose a threat to:

No	Yes	
[x]	[ ]	Public health
[x]	[ ]	National security
[x]	[ ]	Crops and/or livestock
[x]	[ ]	Ecosystems
[x]	[ ]	Any other significant area

Experiments of concern

Does the work involve any of these experiments of concern:

No	Yes	
☒	☐	Demonstrate how to render a vaccine ineffective
☒	☐	Confer resistance to therapeutically useful antibiotics or antiviral agents
☒	☐	Enhance the virulence of a pathogen or render a nonpathogen virulent
☒	☐	Increase transmissibility of a pathogen
☒	☐	Alter the host range of a pathogen
☒	☐	Enable evasion of diagnostic/detection modalities
☒	☐	Enable the weaponization of a biological agent or toxin
☒	☐	Any other potentially harmful combination of experiments and agents

Plants

Seed stocks	Plant leaves were collected from a field experiment (BEF-China) in Xingangshan, in Jiangxi Province, China. From each sampled tree we collected fully developed leaves at three different heights and four different orientations of the crown, resulting in 4,572 leaves.
Novel plant genotypes	No novel plant genotypes were produced.
Authentication	N/A
